# Trends in Abortion- and Contraception-Related Internet Searches After the US Supreme Court Overturned Constitutional Abortion Rights: How Much Do State Laws Matter?

**DOI:** 10.1001/jamahealthforum.2023.0518

**Published:** 2023-04-28

**Authors:** Sumedha Gupta, Brea Perry, Kosali Simon

**Affiliations:** 1 Department of Economics, Indiana University, Indianapolis; 2 Department of Sociology, Indiana University, Bloomington; 3 O’Neill School of Public and Environmental Affairs, Indiana University, Bloomington; 4 National Bureau of Economic Research, Cambridge, Massachusetts

## Abstract

**Importance:**

The US Supreme Court ruling in *Dobbs v Jackson Women’s Health Organization* on June 24, 2022, revealed immediate and distinct differences between states regarding abortion legality. Whether the ruling was associated with population-level changes in seeking information on reproductive health care-related information is unknown.

**Objective:**

To determine whether the US Supreme Court ruling on *Dobbs v Jackson Women’s Health Organization* was associated with increased information seeking for reproductive health care access in the states with immediately effective (trigger and pre-*Roe*) abortion laws vs other states.

**Design, Setting, and Participants:**

This was a retrospective cross-sectional study of nationwide real-time internet search data by state-week from January 1, 2021, through July 16, 2022. Difference-in-difference event study estimates were used to evaluate abortion- and contraception-related internet searches after the Supreme Court draft majority decision was leaked on May 2, 2022, and the final ruling was issued on June 24, 2022, in states immediately affected vs other states. Data analyses were performed from July 18 to January 14, 2022.

**Exposures:**

The Supreme Court’s draft majority decision leaked on May 2, 2022, and the final ruling on *Dobbs v Jackson Women’s Health Organization* on June 24, 2022*.* Preexisting state trigger laws and pre-*Roe* bans that became effective immediately when *Roe* was overturned by the decision on *Dobbs*.

**Main Outcomes and Measures:**

Number of searches per 10 million Google queries in a state-week for terms related to abortion or contraception.

**Results:**

Searches for abortion-related terms increased from 16 302 to 75 746 per 10 million searches per state-week during the weeks before vs after the May 2, 2022, leak of the draft majority decision in states with trigger laws or abortion bans. This was a 42% (95% CI, 24%-59%) higher increase than in states with laws that protect abortion access. Searches for contraception also increased from 56 055 to 82 133 searches per state-week after the ruling in the states with abortion bans, 25% (95% CI, 13%-36%) higher than the increase in states protecting abortion access.

**Conclusions and Relevance:**

The findings of this retrospective cross-sectional study suggest that changes in internet searching for terms related to reproductive health care can capture immediate population-level changes in information-seeking behavior regarding reproductive health care access. These data are critical for shaping health policy discussions.

## Key Points

**Question:**

Were there population-level concerns regarding access to reproductive health care that affected internet-searching behavior after the US Supreme Court ruled on *Dobbs v Jackson Women’s Health Organization*?

**Findings:**

This retrospective cross-sectional study using real-time state-week internet search data found that after the Supreme Court draft decision was leaked to the public on May 2, 2022, internet searches for abortion-related terms increased by 42% more in states with immediately effective abortion bans than in states without “trigger” and pre-*Roe* laws; searches for contraception-related terms also increased by 25% more in these states.

**Meaning:**

The findings of this cross-sectional study suggest that increased internet-based searches for information on reproductive care after the *Dobbs* decision may be an early behavioral indicator of probable gaps in longer-term reproductive health care access in the US.

## Teaser

This cross-sectional study uses nationwide data on real-time state-week internet searches for abortion- and contraception-related information to explore trends associated with the US Supreme Court decision in *Dobbs vs Jackson*.

## Introduction

**Figure 1.**
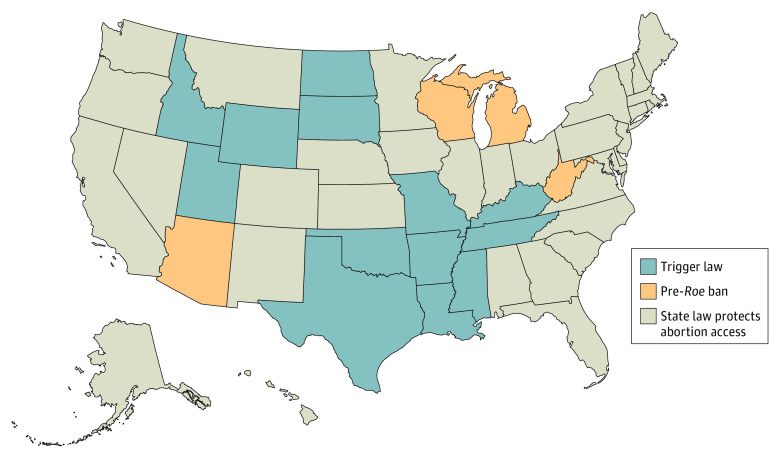
States Abortion Laws, Bans, and Protections After *Roe v Wade* Was Overturned by the US Supreme Court on June 24, 2022 Abortion laws were triggered (became effective immediately) in 13 states (Arkansas, Idaho, Kentucky, Louisiana, Mississippi, Missouri, North Dakota, Oklahoma, South Dakota, Tennessee, Texas, Utah, and Wyoming); and pre-*Roe* abortion bans became effective/enforceable in 4 states (Arizona, Michigan, West Virginia, and Wisconsin). The main analysis considered changes in internet searches using abortion- or contraception-related terms after the leak (May 2, 2022) and after the final ruling (June 24, 2022) in the pooled set of these 17 trigger (blue)/pre-*Roe* ban (orange) states vs states with protected abortion access (beige). Robustness checks considered changes in search activity between states with trigger laws vs states with protected abortion access (eFigures 4 and 5 in Supplement 1); then separately compared states with pre-*R*oe bans vs states with protected abortion access. The data were verified with multiple media sources.^6,7,8^

The US Supreme Court decision on June 24, 2022, in the case of *Dobbs v Jackson Women’s Health Organization* (henceforth, *Dobbs*) ended the federal constitutional right to abortion that had been recognized since its *Roe v Wade* (henceforth, *Roe*) ruling in 1973.^1^ The Court’s majority decision remained substantively the same as its draft opinion, which had been leaked previously to the press on May 2, 2022.^2,3^ The overturning of *Roe* returned abortion regulation to the states. Several states had either pre-*Roe* abortions bans that went back into effect or laws that automatically outlawed abortion with the overturning of *Roe* (henceforth, trigger laws); other states did not.^4,5,6,7,8^ This situation created a quasi-experimental setting for understanding the immediate response of each state’s population to the Constitutional changes limiting access to reproductive health care. Figure 1 summarizes the relevant state policies. A difference-in-differences framework has been successfully used in prior research to evaluate associations with state-level abortion policy change.^9,10,11,12,13^ Although litigation challenging trigger laws is ongoing, legal experts expect that abortion will be criminalized in states with reinstated or triggered abortion bans.^14^

Opinion pieces by experts in leading journals published before the *Dobbs* ruling warned that criminalizing abortion would undermine human rights and harm reproductive health.^15,16,17,18,19,20,21^ In 2020, the maternal mortality rate in the US was 23.8 per 100 000—the highest among all high-income countries.^22^ Even under *Roe*, maternal mortality was higher in states with more restrictive abortion policies.^23^ This pattern raises concern that abortion criminalization will further increase maternal mortality rates and exacerbate mortality disparities between states. Moreover, recruiting and retaining health care professionals will become harder in states that currently allow civil lawsuits against clinicians who are providing support to patients seeking an abortion, further limiting access to reproductive and general health care.^17,18,19,20,21,22,23,24,25^ Reduced access to high-quality, affordable health care may disparately affect the reproductive health of residents of the one-third of the US counties that are *maternity care deserts* (lacking quality and/or affordable pre- and postnatal care). These counties, which are disproportionately in states that did not expand Medicaid, are politically conservative^24^ and have more restrictive abortion policies.

*Dobbs* may also widen disparities between Black and White maternal and infant morbidity and mortality rates.^21^ Maternal mortality among Black women (55.3 deaths per 100 000 live births) is nearly 3 times the rate among White women (19.1 deaths per 100 000 births).^24^ Studies using quasi-experimental research designs exploiting state variations in policy stringency show that abortion legalization was associated with reductions in births (4%-11%), teen pregnancy (34%), and teen marriage (20%), with the largest reductions seen among Black adolescents.^25,26^ However, in absence of real-time national surveys or health care utilization data, there has been little evidence to signal how reproductive health care access and potential behavioral responses regarding contraception may have changed since the *Dobbs* ruling.^27^ An aim of the present study was to contribute to the available literature on this topic.

Prior studies have used internet search data to assess immediate responses to policy changes and public health events.^28,29^ Policies criminalizing abortion affect decision-making and behavior among individuals who are sexually active and/or are of reproductive age. Information seeking through internet searches may reflect concerns regarding constrained reproductive health care access among individuals with or at-risk for unintended pregnancy.^2^ A 2010 study^30^ found that state abortion restrictions were positively associated with the internet search volumes for the term “abortion” and negatively associated with the local abortion rate, patterns consistent with individuals seeking abortion services outside of their local area after restrictions were enacted.

Using data from Google search trends, this study aimed to provide a timely analysis of how the *Dobbs* ruling affected information seeking for reproductive health care access, which may foreshadow potential consequences for reproductive health.^2,26^ Exploiting the differential legal repercussions of *Dobbs* on abortion criminalization across states, we used a difference-in-differences study design to evaluate how online information-seeking behavior regarding abortion and contraception care changed in response to the leaked draft of the Supreme Court decision and the release of the final version of the ruling. We compared states whose policies did not change after *Dobbs* with states whose policies did change (ie, those with trigger or pre-*Roe* bans).

## Methods

This study used deidentified secondary data and, therefore, was not subject to review by institutional review board of Indiana University, nor was informed consent necessary. The study followed the Strengthening the Reporting of Observational Studies in Epidemiology (STROBE) guidelines for cross-sectional studies.

### Data Collection

The analysis used balanced, weekly panel data on the intensity of internet searches for terms pertaining to reproductive health within all 50 states and Washington, DC (henceforth, DC), from January 1, 2021, through July 16, 2022 (n = 51 states/district × 80 weeks). Using a restricted-access Google Health Trends Application Program Interface research account, we obtained data that captured 92.1% of the search engine market as of February 2021,^31,32^ with more than 8.1 billion unique monthly visitors as of November 2022.^33^ Demographic information on the internet users who searched for reproductive health-related data were not collected.

### Study Measures

The main outcome measured was the number of searches per 10 million total Google queries in a state-week for terms related to abortion and contraception and/or safe sex (henceforth, contraception) during the study period. Abortion search terms included “abortion,” “abortion pill,” and “plan B,” plus the 2 most commonly used abortion-inducing medications, “mifepristone” and “misoprostol.” Contraception search terms included several general contraception terms (eg, “contraceptives,” “safe sex”), as well as specific terms for contraceptives that are short acting (eg, “pills,” “condoms”), long acting (eg, “implant,” “intrauterine device or IUD,” “Nexplanon”), and permanent (eg, tubal ligation, vasectomy). We merged the state-week search intensity data with state abortion law information from multiple sources.^6,34^

We distinguished between the 17 states where the legal conditions changed immediately after the June 24, 2022, ruling—13 with trigger laws and 4 with pre-*Roe* bans (Figure 1)—and the states and district where they did not.^10^ We considered enforceable abortion policy changes that occurred after the ruling. However, given the 3071 protests from May 2 to July 1, 2022,^7^ widespread political mobilization,^8^ and the unsettling repercussions on reproductive health care in some states^30^ immediately after the leaking of the draft decision on May 2, 2022, and before the official ruling was announced, we evaluated data trends before the leak.

There are growing concerns that *Dobbs* may spark reproductive policy changes in “red*”* states, which have a pro-life majority and which we defined as having continuously elected a Republican governor from January 2015 through July 2022. None of the trigger or pre-*Roe* ban states were “blue,*”* defined as having continuously elected a Democratic governor during the same period. Ten red states—Alabama, Florida, Georgia, Iowa, Indiana, Massachusetts, Maryland, Nebraska, Ohio, and South Carolina—also did not have trigger laws or pre-*Roe* bans—although they could soon be affected. Thus, given the policy uncertainty in the traditionally Republican states, we used predetermined secondary analyses to evaluate changes according to the political affiliation of the state’s governor. Details on the political classification of US states are available in eTable 1 in Supplement 1.

### Statistical Analyses

Unadjusted trends in internet queries in the days surrounding the US Supreme Court’s *Dobbs* ruling were evaluated by plotting the daily share of total Google queries from each of the 50 states and DC corresponding to either abortion- or the contraception-related terms. The main regression fits a Poisson model in an event study framework,^35^ capturing internet searches after May 2, 2022 (leaked draft), in the 17 states that would be immediately affected by their trigger or pre-*Roe* bans vs the other states (refer to the eMethods in Supplement 1 for more regression details, including sensitivity checks). We also evaluated changes in search intensity at 7 weeks after the official ruling vs the week before the leak. All regressions included state, year, and year-of-the week fixed-effects to control for time-invariant state differences, seasonality, and secular changes in reproductive health-related search behavior. Standard errors were adjusted for heteroskedasticity and clustering at the state level to account for within-state correlations over time. Poisson coefficients with 95% CIs were reported for the 10 weeks before May 2, 2022 (draft leak), and for the subsequent 9 weeks. The estimates capture the differential percentage change in search intensity during each week vs the week before the leak (reference week with normalized value of 0) in states with trigger laws or pre-*Roe* bans compared with other states (eFigures 1-8 in Supplement 1). All analyses were performed using Stata, version 17.0 (StataCorp). Statistical tests were 2-tailed and *P* values < .05 were considered statistically significant. Data analyses were performed from July 18 to January 14, 2022.

## Results

**Figure 2.**
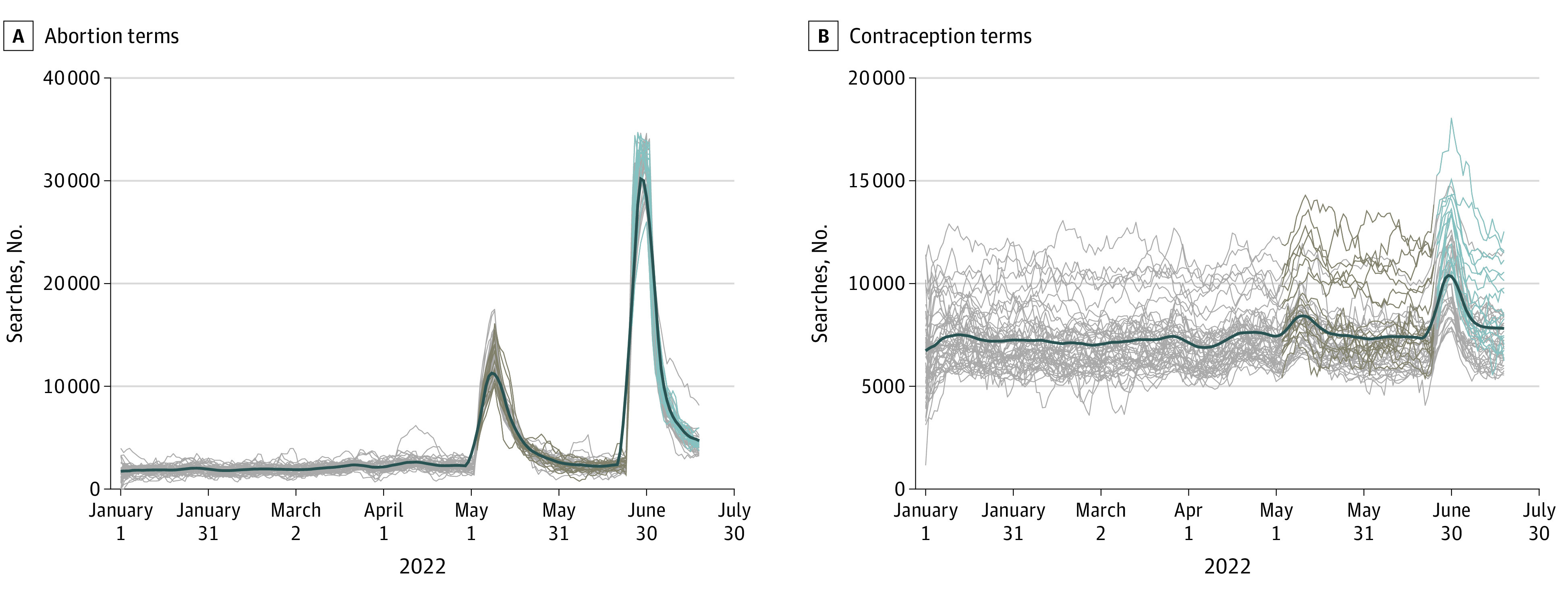
Internet Searching Before and After the Leaked Draft Decision (May 2, 2022) and the Final Ruling (June 24, 2022) by the US Supreme Court on *Dobbs* Outcome variables measured the daily share of all Google queries in a state using abortion- or contraception-related terms × 10 million rounded to the nearest integer. Gray lines represent states and become brown for states with trigger laws or pre-*Roe* bans and then blue after the official ruling (see Figure 1 for classification). The thick black line represents a “smoothed” 7-day moving average for all states capturing the national trend. Based on data from Google Health Trends, January 1-July 16, 2022.

Figure 2 presents the national and state-by-state unadjusted internet search trends for abortion- and contraception-related terms. We noted sharp increases in internet searches for both abortion and contraception terms after the leak, and then more so after the official ruling 7 weeks later. After the leak and ruling, searches for abortion terms appeared to increase equally dramatically in immediately affected (trigger/pre-*Roe* ban) as well as other states (Figure 2A). Similarly, although searches for contraception terms spiked in all states, the associated increase was particularly large in the states with trigger laws or pre-*Roe* ban (Figure 2B).

**Figure 3.**
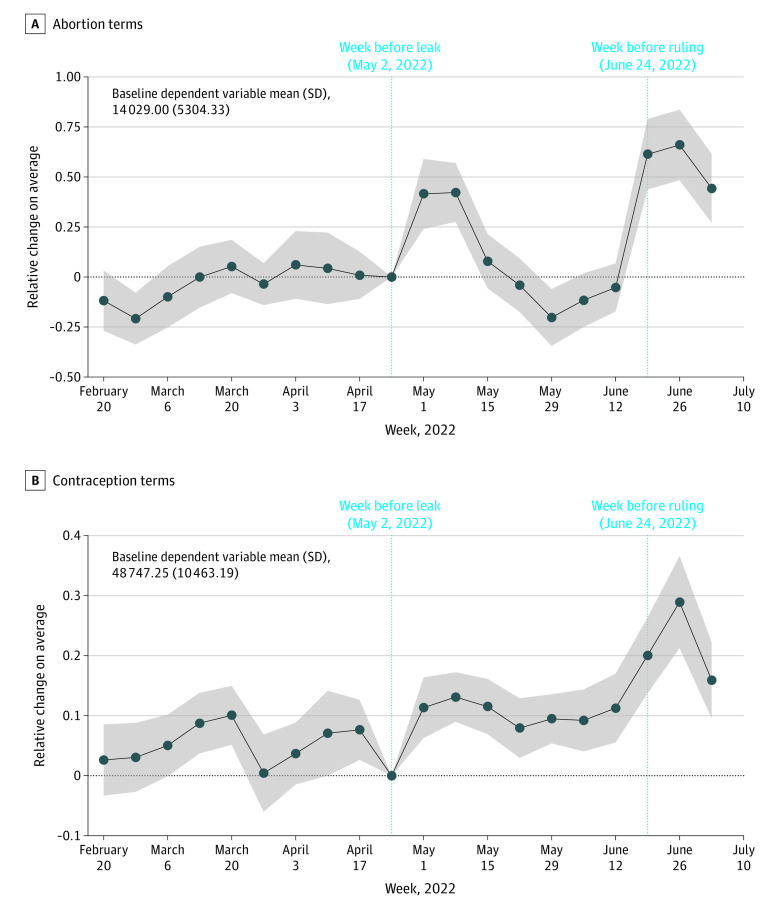
Time-Varying Effects on Internet Searching of Abortion- or Contraceptive-Related Terms After the *Dobbs* Leaked Draft and Official Ruling in States With Abortion Trigger Laws or Pre-*Roe* Bans Relative to States With Protected Abortion Access The week before treatment (leak) is the reference (coefficient value normalized to 0) depicted as the light blue dotted vertical line. The week before the official ruling on June 24, 2022, is depicted as the second light blue dotted vertical line. Solid lines are the estimated coefficients with gray bands representing the 95% CIs in the Poisson model (differences in log expected counts of search relative to the reference week). Additional data are available in (eFigures 1 and 2 in Supplement 1).

Figure 3A depicts event study estimates of the relative percentage increases in abortion-related internet searches during the weeks before and after the leak (May 2, 2022) and the official ruling (June 24, 2022) (eTable 2 in Supplement 1 provides search counts per state-week for January 1-July 16, 2022, by state abortion law status). Specifically, in states with trigger or pre-*Roe* laws, searches for abortion terms increased from 16 302 searches per 10 million per state-week (M/state-wk) before May 2, 2022, to 75 746 searches, with a natural logarithm (ln) calculation of ln(75 746/16 302) indicating 1.53 times more searches during the week immediately after that date. Concurrently, in states with laws protecting abortion access, the search intensity increased from 20 023 searches per 10 M/state-wk to 61 426 searches; that is, 1.11 times more searches calculated as ln(61 426/20 023). The difference implies a 42% (95% CI, 24%-59%) greater increase in states with trigger laws or pre-*Roe* bans vs states with protected abortion access (ie, differential increase, 1.53 − 1.11 = 0.42). During the next 5 weeks, the search intensity for abortion terms returned to preleak levels. Then, during the week of June 24, 2022 (the ruling), searches increased sharply to 150 602 per 10 M/state-wk in states with trigger laws or pre-*Roe* bans (2.22 times more searches) and to 100 183 searches in other states (1.61 times more searches), implying a 61% (95% CI, 44%-79%) greater increase in search intensity in states with trigger laws or pre-*Roe* bans (incremental increase, 2.22 − 1.61 = 0.61). Thereafter and through the last week of the study period (July 16, 2022), the average search intensity among states with trigger or pre-*Roe* bans remained approximately 44% (95% CI, 27%-62%) greater than baseline and greater than in states with protected abortion access.

There was no observable trend in search behavior during the weeks before the leak (ie, no pretrend), suggesting that the leak may indeed have been associated with heightened collective attention to the threat of abortion criminalization. Changes were concentrated in broad search terms, such as “abortion” and “plan B,” whereas searches for specific abortion medications remained relatively stable throughout the study period (eFigure 1 and eTable 4 in Supplement 1).

Figure 3B presents event studies for contraception-related terms (eTable 2 in Supplement 1 provides search counts per state-week for January 1-July 16, 2022, by state abortion law status). These searches started trending slightly upwards just before the leak, increasing by 11% (95% CI, 6%-16%) more in states with trigger or pre-*Roe* bans (56 055 to 64 808 searches per 10 M/state-wk) than in other states (49 669 to 51 275) in the weeks before and after the leak. Immediately after the official ruling, searches spiked with nearly 25% (95% CI, 13%-36%) more searches in states with trigger laws or pre-*Roe* bans (82 133 searches than in other states (54 492 searches). Changes were apparent in the increased searches for long-term contraception (eg, implants, 28% [95% CI, 15%-36%] and IUDs, 26% [95% CI, 17%-36%]) and permanent contraception (eg, tubal ligation, 41% [95% CI, 14%-69%] and vasectomy, 67% [95% CI, 43%-90%] greater than baseline numbers); both remained significantly higher than the previously flat baseline levels through the end of the study period (eFigure 2 and eTable 5 in Supplement 1 provide the adjusted search counts per state-week).

**Figure 4.**
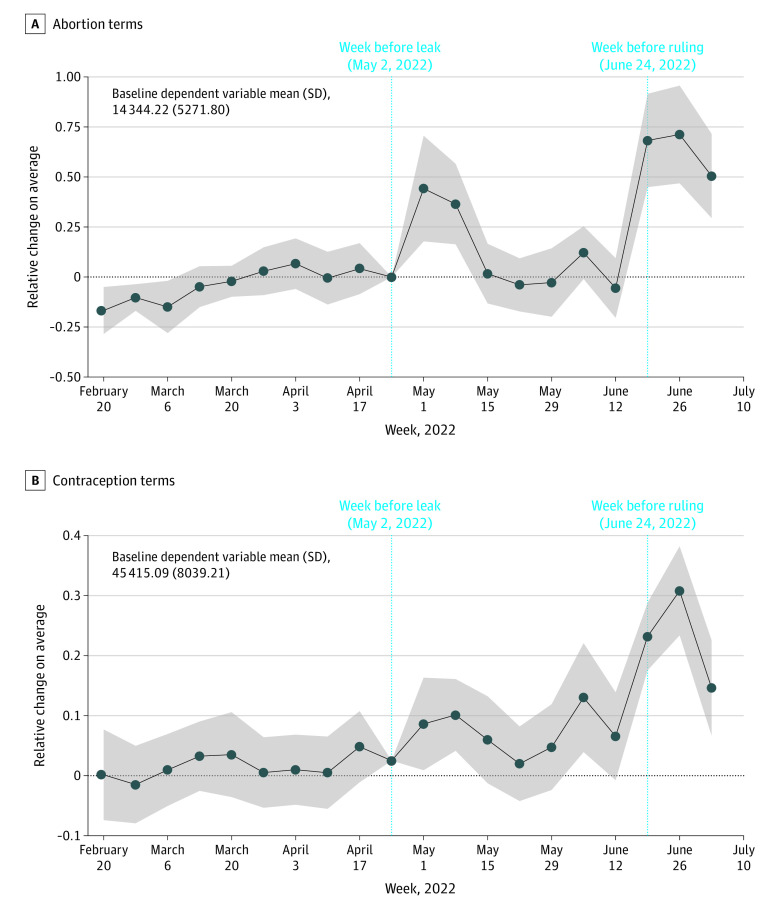
Time-Varying Effects on Internet Searching Using Abortion- or Contraceptive-Related Terms After *Dobbs* Ruling in States With No Trigger Laws or Reinstated Abortion Bans in States With Established Republican Governors (Red States) Relative to Democratic Governors (Blue States) Based on states stratified by the governor’s political affiliation from 2015-2022. Historically blue states (California, Colorado, Connecticut, Delaware, Minnesota, New York, Oregon, Pennsylvania, Rhode Island, Washington, and the District of Columbia) and red states (Alabama, Arkansas, Arizona, Florida, Georgia, Iowa, Idaho, Indiana, Massachusetts, Maryland, Mississippi, North Dakota, Nebraska, Ohio, Oklahoma, South Carolina, South Dakota, Tennessee, Texas, Utah, and Wyoming) were included; the remaining 18 states were excluded (Alaska, Hawaii, Kansas, Kentucky, Louisiana, Maine, Michigan, Missouri, North Carolina, New Hampshire, New Jersey, New Mexico, Nevada, Virginia, Vermont, Wisconsin, and West Virginia). Additional data are available in eTable 1 in Supplement 1). The week before treatment (leak) is the reference (coefficient value normalized to 0) depicted as the light blue dotted vertical bar. The week before the official ruling on June 24, 2022, is depicted as the second light blue dotted vertical line. Solid lines are the estimated coefficients with gray bands representing the 95% CIs in the Poisson model (differences in log expected counts of search relative to the reference week).

For the subsample of states without trigger laws or pre-*Roe* bans, Figure 4 shows event-study−based comparisons of changes in internet search terms after the *Dobbs* leak in red vs blue states (eTable 3 in Supplement 1). Figure 4A indicates that during the week after the leak, even red states without trigger laws or pre-*Roe* bans experienced search increases, from 15 791 to 73 587 per 10 M/state-wk, a 44% (95% CI, 18%-71%) greater increase in abortion-related searches vs blue states (20 881 to 62 560 searches). After declining to preleak levels, which were similar to those of blue states during the next 4 weeks, searches for abortion terms in red states without trigger laws or pre-*Roe* bans increased to 141491 searches per 10 M/state-wk, 68% (95% CI, 45%-92%) more than the increase to 94 568 in blue states without trigger or pre-*Roe* bans. Again, we noticed no significant pretrends before the leak, suggesting that the leak amplified information seeking in response to the threat of abortion criminalization. Figure 4B shows that searches for contraception terms in red states without trigger laws or pre-*Roe* bans mimicked those of states with trigger laws or pre-*Roe* bans; that is, the trend was moving slightly upwards just before the leak. After the official ruling, there was a sharp increase to 65 609 searches per 10 M/state-wk in red states, which was a 23% (95% CI, 18%-29%) greater increase than in blue states (48 005 searches).

eFigure 3 in Supplement 1 reiterates that the *Dobbs’* repercussions may go beyond the states with trigger laws or pre-*Roe* bans (absolute search counts are available in eTable 6 in Supplement 1). We found heightened interest in abortion and contraception terms in *all* red states. Searches for abortion-related terms increased from 15 331 to 81 135 per 10 M/state-wk in red states, a 60% (95% CI, 32%-87%) greater increase than in blue states (22 018 to 55 204 searches), during the week of the leak. Then, after the ruling, searches increased to 143 182 searches per 10 M/state-wk, an 88% (95% CI, 60% to 115%) greater increase than in blue states (81 041 searches). Similarly, searches for contraception-related terms were concentrated in red states (irrespective of trigger/pre-*Roe* ban status) and started trending upwards just before the leak; after the ruling, they increased from 16 154 to 139 434 searches per 10 M/state-wk, a 30% (95% CI, 23%-38%) greater increase than in blue state increase (45 596 to 47 054 searches; details available in eFigure 3B in Supplement 1).

### Robustness Checks

First, we investigated whether the information-seeking response to the *Dobbs* leak differed in the states with trigger laws (eFigure 4 and eTable 7 in Supplement 1 for counts/state-week) or pre-*Roe* bans (eFigure 5 and eTable 8 in Supplement 1 for intensity/state-week). We found immediate relative increases after the leak in abortion-related searches in states with trigger laws (16 601 increasing to 74 087 searches per 10 M/state-wk vs 19 697 to 62 504 searches in other states, implying a 34% [95% CI, 16%-52%] higher increase) and in states with pre-*Roe* bans (15 331 to 81 135 searches vs 19 284 to 65 722 searches in other states, implying a 44% [95% CI, 19%-69%] higher increase), with a 10 percentage point (pp) higher relative increase in those with pre-*Roe* bans. Similarly, after the ruling, the increase in abortion-related searches was 16 pp higher in states with pre-*Roe* bans (167 705 vs 108 998 searches per 10 M/state-wk, a 66% [95% CI, 52%-80%] higher increase) than in states with trigger laws (145 340 vs 102 761 searches, a 51% [95% CI, 33%-71%] higher increase). In all instances, we found no observable pretrend in search behavior, suggesting that the leak amplified the threat of abortion criminalization in states with trigger laws or pre-*Roe* bans. Increased search activity for contraception-related terms after the ruling was also observed in states with trigger laws (57 701 to 65 735 searches per 10 M/state-wk vs 49 654 to 51 398 searches in other states, a 10% [95% CI, 4%-16%] higher increase) or pre-*Roe* bans (50 707 to 61 797 searches vs 49 658 to 51 579 in other states, a 16% [95% CI, 13%-19%] higher increase).

Second, we evaluated whether the changes observed in internet searching behavior in states with trigger laws or pre-*Roe* bans were prompted by the states that criminalized abortion early, ie, before the *Dobbs* ruling. In May 2021, Texas became the first state to ban abortion beyond 6 weeks of gestation,^36^ directly challenging the Supreme Court’s 1973 *Roe* v *Wade* ruling. Subsequently, on May 25, 2022, Oklahoma implemented the strictest antiabortion law in the US, banning abortions from the point of fertilization and allowing private citizens to sue abortion clinicians who “knowingly perform or induce an abortion on a pregnant a pregnant person, on May 25, 2022, prior to the US Supreme Court’s ruling in Dobbs on June 24, 2022.”^37^ Again, this was before the Supreme Court’s official ruling on *Dobbs*. Therefore, in the first check, we re-estimated the main model, excluding Texas and Oklahoma. As eFigure 6 in Supplement 1 indicates, the changes in abortion- and contraception-related search activity among the remaining states with trigger laws or pre-*Roe* bans were statistically no different from our main findings of 42% (95% CI, 25%-61%) and 64% (95% CI, 47%-82%) relative increases in abortion-related searches after the leak and the ruling, respectively, with no significant pretrend in search behavior (eTable 9 in Supplement 1 provides search intensity data per state-week). Also comparable to the main results, contraception-related searches increased by 28% (95% CI, 21%-29%) after the ruling in states with trigger laws or pre-*Roe* bans, excluding Texas and Oklahoma. As a second placebo check, we re-estimated the main model using only data from Texas and Oklahoma, where abortion bans had been implemented before the *Dobbs* decision (eFigure 7 and eTable 10 in Supplement 1). The leak on May 2, 2022, of the draft majority decision was still associated with a smaller but statistically significant increase in search intensity for contraception-related terms.

Third, we evaluated whether search behavior changed only after the final ruling (not after the leak, as in the main specification). As eFigure 8 in Supplement 1 indicates, there were significantly diverging trends in both abortion- and contraception-related internet searches in states with trigger laws or pre-*Roe* bans compared with other states during the weeks coinciding with the leak and before the official ruling (eTable 11 in Supplement 1 provides the search intensity per state-week data). Therefore, we did not find evidence to support the idea that changes in reproductive health-related information-seeking behavior occurred only after the ruling.

Lastly, because Google trends data do not indicate search intentions, we evaluated whether the amplified internet search activity in affected states was for specific reproductive care services (rather than general information seeking) by estimating changes in queries for “Planned Parenthood” after the leak in states with trigger laws or pre-*Roe* bans vs other states. As shown in eFigure 9 in Supplement 1, the relative increase in searches for Planned Parenthood in states with trigger laws or pre-*Roe* bans after the leak and after the ruling, respectively, were 47% (95% CI, 31%-63%; 2685 to 6470 searches per 10 M/state-wk vs 4410 to 6644 in other states) and 60% (95% CI, 42%-78%; 3238 to 9112 vs 4555 to 8207 in other states) (eTable 12 in Supplement 1 presents the search intensity per state-week). Together with the large relative increases in specific search terms, such as “plan B” and “Planned Parenthood,” these findings suggest that the increased abortion- and contraception-related internet search activity was at least partially associated with specific reproductive health services provided by organizations, such as Planned Parenthood, not solely general information seeking.

## Discussion

To what extent do these study findings showing more internet searches for reproductive health services reflect all information seeking on this topic? These findings suggest the possibility that individuals experiencing restricted in-state abortion services may have used internet searches with terms such as Planned Parenthood, plan B, tubal ligation, and vasectomy to locate abortion services in neighboring states or to find information on the safety, effectiveness, and availability of abortion pills for self-managed abortion.^38^ However, it is also possible that individuals in states with restricted abortion access were more likely to seek abortion information through local resources, such as an obstetrician-gynecologist or a primary care practitioner. In that case, our results on greater internet searches in restricted states would have underestimated the actual increase. Alternatively, individuals seeking abortion or contraception in blue states (where continued access to abortion services are available) would have been less likely to search using abortion-related terms than their peers in red states would have been. This interpretation is consistent with evidence that because of state abortion restrictions, individuals travel to other states to obtain procedures^9,39,40^ and that internet searches for “abortion” occur disproportionately in locations with restricted abortion access and fewer abortions performed.^34^

These study findings may foreshadow heightened maternal risk associated with delaying abortion to higher gestational age because it requires traveling to a state with abortion access, psychological distress, and self-induced abortion.^39^ Traveling farther to obtain abortion services may also be associated with longer periods of lost wages and higher childcare costs and travel expenses, imposing previously reported longer-term financial repercussions for individuals denied abortions locally, with secondary effects on infant health and children’s living conditions.^13,14,15^ Because individuals seeking abortion services are disproportionately from low-income and/or racial and ethnic minority groups, the adverse consequences of traveling to another state for abortion access may exacerbate existing health disparities.^40^

Although we found larger relative increases in internet searches for abortion-related terms, the analysis also identified a heightened number of searches for contraception-related terms in states with trigger laws or pre-*Roe* bans. This finding suggests that the threat to reproductive autonomy may have been interpreted more broadly, extending to fears that contraception could become limited in red states. Alternatively, the higher number of searches for contraceptives, especially long-term and permanent contraceptive methods, may indicate that *Dobbs* has changed individual contraception decisions. This suggestion aligns with anecdotal evidence reported by National Public Radio^41^ and other news outlets that there is a growing interest in sterilizations, especially among younger individuals, since the *Dobbs* ruling.

### Limitations

This study had some limitations. First, we did not capture actual provision of care. However, internet search behavior has been shown to be indicative of abortion policy-induced population concerns and may be useful as surveillance measures capturing immediate responses to policy before the availability of traditional survey and health care utilization data.^30,39^ Also, we were unable to determine the search intentions of users to ascertain what portion of searches reflected information seeking for reproductive health services or information on the ruling itself. However, our findings of heightened search intensity for terms such as “plan B” and “tubal ligation” suggest that the increased abortion- and contraception-related internet search activity may partly reflect individuals searching for specific reproductive health services, beyond any general political interest. Finally, these findings captured only the immediate responses to the Supreme Court’s majority decision leak and ruling and did not track the evolving state-by-state law changes. Many state courts are blocking implementation of abortion bans, creating ambiguity in their effective dates. However, these findings do capture the likely repercussions of the Supreme Court’s ruling on reproductive care, with clinicians now facing potential civil lawsuits and criminal charges for provision of certain types of care, particularly in red states and states with trigger laws or pre-*Roe* abortion bans.^42^

## Conclusions

This retrospective cross-sectional study using nationwide data on real-time internet search activity documented population-level increases in reproductive health care-related internet search behavior after the Supreme Court’s ruling on *Dobbs* v *Jackson Women’s Health Organization* in states affected by trigger laws or pre-*Roe* bans compared with states with protected abortion access. Our findings reflect early heightened collective attention both to immediate state-level abortion bans and to the threat of abortion criminalization in traditionally red states. These changes in reproductive health-related information-seeking behavior may be interpreted as early indicators of probable longer-term outcomes and could be critical for shaping ongoing policy discussions. Moreover, to the extent that internet search trends reflect the pursuit of specific reproductive health services, the study results reiterate that internet-based information dissemination, including information on where individuals can legally and safely obtain reproductive care or telemedicine consultations with health care professionals,^39^ may serve as a viable harm-reduction strategy.

## Notes

Supplement 1. **eMethods.** Description of Multivariable Regression Approach **eTable 1.** Mapping of states with laws that ban most or all abortions (trigger and pre-Roe ban states) and Democrat blue and Republican red states. **eTable 2. **Estimated number of searches for abortion and contraception terms in trigger/ pre-Roe abortion ban states and other states with laws protecting abortion access between January 2, 2022 and July 16, 2022. **eTable 3. **Estimated number of searches for abortion and contraception terms in non- trigger/pre-Roe ban “red” states vs “blue” states between January 2, 2022 and July 16, 2022. **eTable 4. **Estimated number of searches for specific abortion terms in trigger/ pre-Roe abortion ban states and other states with laws protecting abortion access between January 2, 2022 and July 16, 2022. **eTable 5.** Estimated number of searches for specific contraception terms in trigger/ pre-Roe abortion ban states and other states with laws protecting abortion access between January 2, 2022 and July 16, 2022. **eTable 6. **Estimated number of searches for abortion and contraception terms in “red” versus “blue” states between January 2, 2022 and July 16, 2022. **eTable 7.** Estimated number of searches for abortion and contraception terms in immediately impacted trigger states and other states with laws protecting abortion access between January 2, 2022 and July 16, 2022. **eTable 8.** Estimated number of searches for abortion and contraception terms in pre-Roe abortion ban states and other states with laws protecting abortion access between January 2, 2022 and July 16, 2022. **eTable 9.** Estimated number of searches for abortion and contraception terms in Trigger/ pre-Roe abortion ban states, excluding early adopters Texas and Oklahoma, and other states with laws protecting abortion access between January 2, 2022 and July 16, 2022. **eTable 10. **Estimated number of searches for abortion and contraception terms in early adopters Texas and Oklahoma, and other states with laws protecting abortion access between January 2, 2022 and July 16, 2022. **eTable 11.** Estimated number of searches for abortion and contraception terms in trigger/ pre-Roe abortion ban states and other states with laws protecting abortion access between January 2, 2022 and July 16, 2022. **eTable 12.** Estimated number of searches for “Planned Parenthood” in trigger/ pre-Roe abortion ban states and other states with laws protecting abortion access between January 2, 2022 and July 16, 2022. **eFigure 1.** Abortion search term details. **eFigure 2. **Contraception term details - Queries for short-acting, long-acting and permanent forms of contraception. **eFigure 3. **Time-varying effects of the leak of the U.S. Supreme Court’s draft majority opinion in Red states compared to Blue states. **eFigure 4.** Time-varying effects of the leak of the U.S. Supreme Court’s draft majority opinion in Dobbs on May 2, 2022, in trigger states vs States where state law protects abortion access. **eFigure 5.** Time-varying effects of the leak of the U.S. Supreme Court’s draft majority opinion in Dobbs v Jackson Women’s Health Organization on May 2, 2022, in pre-Roe states vs States where state law protects abortion access. **eFigure 6. **Time-varying effects of the leak of the U.S. Supreme Court’s draft majority opinion in Dobbs on May 2, 2022, in trigger/pre-Roe ban states, excluding early adopters Texas and Oklahoma, vs States where state law protects abortion access. **eFigure 7.** Time-varying effects of the leak of the U.S. Supreme Court’s draft majority opinion in Dobbs on May 2, 2022, in early adopters Texas and Oklahoma, vs States where state law protects abortion access. **eFigure 8.** Time-varying effects of the U.S. Supreme Court’s ruling in Dobbs v Jackson Women’s Health Organization on June 24, 2022, in trigger and pre-Roe states vs States where state law protects abortion access. **eFigure 9.** Time-varying effects of the Dobbs Leak and Official Ruling on internet searches for ‘Planned Parenthood’, in trigger/pre-Roe ban states vs in states with laws protecting abortion access.

Supplement 2.
Data Sharing Statement
