# Supplementary material for: Trends in Abortion- and Contraception-Related Internet Searches After the US Supreme Court Overturned Constitutional Abortion Rights: How Much Do State Laws Matter?
Source: JAMA Health Forum. 2023 Apr 28;4(4):e230518. doi: 10.1001/jamahealthforum.2023.0518 (PMC10148201; doi:10.1001/jamahealthforum.2023.0518)
Supplement: Supplement 1. — eMethods. Description of Multivariable Regression Approach eTable 1. Mapping of states with laws that ban most or all abortions (trigger and pre-Roe ban states) and Democrat blue and Republican red states. eTable 2. Estimated number of searches for abortion and contraception terms in trigger/ pre-Roe abortion ban states and other states with laws protecting abortion access between January 2, 2022 and July 16, 2022. eTable 3. Estimated number of searches for abortion and contraception terms in non- trigger/pre-Roe ban “red” states vs “blue” states between January 2, 2022 and July 16, 2022. eTable 4. Estimated number of searches for specific abortion terms in trigger/ pre-Roe abortion ban states and other states with laws protecting abortion access between January 2, 2022 and July 16, 2022. eTable 5. Estimated number of searches for specific contraception terms in trigger/ pre-Roe abortion ban states and other states with laws protecting abortion access between January 2, 2022 and July 16, 2022. eTable 6. Estimated number of searches for abortion and contraception terms in “red” versus “blue” states between January 2, 2022 and July 16, 2022. eTable 7. Estimated number of searches for abortion and contraception terms in immediately impacted trigger states and other states with laws protecting abortion access between January 2, 2022 and July 16, 2022. eTable 8. Estimated number of searches for abortion and contraception terms in pre-Roe abortion ban states and other states with laws protecting abortion access between January 2, 2022 and July 16, 2022. eTable 9. Estimated number of searches for abortion and contraception terms in Trigger/ pre-Roe abortion ban states, excluding early adopters Texas and Oklahoma, and other states with laws protecting abortion access between January 2, 2022 and July 16, 2022. eTable 10. Estimated number of searches for abortion and contraception terms in early adopters Texas and Oklahoma, and other states with laws protecting abortion acces [file jamahealthforum-e230518-s001.pdf]

Supplemental Online Content

Gupta S, Perry B, Simon K. Trends in abortion- and contraception-related internet searches after the US supreme court overturned constitutional abortion rights: how much do state laws matter? *JAMA Health Forum*. 2023;4(4):e230518. doi:10.1001/jamahealthforum.2023.0518

eMethods. Description of Multivariable Regression Approach

eTable 1. Mapping of states with laws that ban most or all abortions (trigger and pre-Roe ban states) and Democrat blue and Republican red states.

eTable 2. Estimated number of searches for abortion and contraception terms in trigger/ pre-Roe abortion ban states and other states with laws protecting abortion access between January 2, 2022 and July 16, 2022.

eTable 3. Estimated number of searches for abortion and contraception terms in non- trigger/pre-Roe ban "red" states vs "blue" states between January 2, 2022 and July 16, 2022.

eTable 4. Estimated number of searches for specific abortion terms in trigger/ pre-Roe abortion ban states and other states with laws protecting abortion access between January 2, 2022 and July 16, 2022.

eTable 5. Estimated number of searches for specific contraception terms in trigger/ pre-Roe abortion ban states and other states with laws protecting abortion access between January 2, 2022 and July 16, 2022.

eTable 6. Estimated number of searches for abortion and contraception terms in "red" versus "blue" states between January 2, 2022 and July 16, 2022.

eTable 7. Estimated number of searches for abortion and contraception terms in immediately impacted trigger states and other states with laws protecting abortion access between January 2, 2022 and July 16, 2022.

eTable 8. Estimated number of searches for abortion and contraception terms in pre-Roe abortion ban states and other states with laws protecting abortion access between January 2, 2022 and July 16, 2022.

eTable 9. Estimated number of searches for abortion and contraception terms in Trigger/ pre-Roe abortion ban states, excluding early adopters Texas and Oklahoma, and other states with laws protecting abortion access between January 2, 2022 and July 16, 2022.

© 2023 Gupta et al. *JAMA Health Forum.*

eTable 10. Estimated number of searches for abortion and contraception terms in early adopters Texas and Oklahoma, and other states with laws protecting abortion access between January 2, 2022 and July 16, 2022.

eTable 11. Estimated number of searches for abortion and contraception terms in trigger/ pre-Roe abortion ban states and other states with laws protecting abortion access between January 2, 2022 and July 16, 2022.

eTable 12. Estimated number of searches for "Planned Parenthood" in trigger/ pre-Roe abortion ban states and other states with laws protecting abortion access between January 2, 2022 and July 16, 2022.

eFigure 1. Abortion search term details.

eFigure 2. Contraception term details - Queries for short-acting, long-acting and permanent forms of contraception.

eFigure 3. Time-varying effects of the leak of the U.S. Supreme Court's draft majority opinion in Red states compared to Blue states.

eFigure 4. Time-varying effects of the leak of the U.S. Supreme Court's draft majority opinion in Dobbs on May 2, 2022, in trigger states vs States where state law protects abortion access. Treated sample includes only 'trigger' states; pre-Roe ban states are excluded from the analysis.

eFigure 5. Time-varying effects of the leak of the U.S. Supreme Court's draft majority opinion in Dobbs v Jackson Women's Health Organization on May 2, 2022, in pre-Roe states vs States where state law protects abortion access. Treated sample includes only the four 'pre-Roe' states; 'trigger' states are excluded from the analysis.

eFigure 6. Time-varying effects of the leak of the U.S. Supreme Court's draft majority opinion in Dobbs on May 2, 2022, in trigger/pre-Roe ban states, excluding early adopters Texas and Oklahoma, vs States where state law protects abortion access.

eFigure 7. Time-varying effects of the leak of the U.S. Supreme Court's draft majority opinion in Dobbs on May 2, 2022, in early adopters Texas and Oklahoma, vs States where state law protects abortion access.

eFigure 8. Time-varying effects of the U.S. Supreme Court's ruling in Dobbs v Jackson Women's Health Organization on June 24, 2022, in trigger and pre-Roe states vs States where state law protects abortion access.

eFigure 9. Time-varying effects of the Dobbs Leak and Official Ruling on internet searches for 'Planned Parenthood', in trigger/pre-Roe ban states vs in states with laws protecting abortion access

© 2023 Gupta et al. *JAMA Health Forum.*

This supplemental material has been provided by the authors to give readers additional information about their work.

© 2023 Gupta et al. *JAMA Health Forum.*

eMethods. Description of Multivariable Regression Approach

The objective of the analysis was to assess the changes in reproductive health related information seeking behavior, indicative of population-level concerns about continued access to reproductive health following the US Supreme Court's Dobbs ruling and measured by the daily share of all Google queries in a state that correspond to terms for (A) abortion, and (B) contraception (refer to Methods section of manuscript for individual search terms included in each category). As any one term represents only a small fraction of all Google searches in a state on a given day, we multiply the shares by 10 million and round to the nearest integer to make the measure more interpretable.

We model the expected number of searches per 10 million in state *s*, in year *y*, and week *w* as:

$$y_{syw} = e^{\left(\beta_0 + \sum_{a=-10}^{9} \beta_a Dobbs_a + \theta_s + \theta_y + \theta_w + \varepsilon_{st}\right)}$$

Where $Dobbs_a$ is an indicator variable set to 1 if the Dobbs ruling occurred 'a' weeks ago. The reference category was 1 week prior to the Dobbs leak, and we created single indicators $Dobbs_{10}$ for any a>=9, and $Dobbs_{-1}$ for any a<=-10. All regressions control for time invariant state differences (θ_s), seasonality (θ_w) and secular changes (θ_y) in abortion and contraception related search behavior. Standard errors are adjusted for heteroskedasticity and clustering at the state level to account for within state correlations over time. The exponential conditional mean function is an appealing approximation here because it accommodates the restriction that the conditional mean value of Google searches is non-negative in all weeks. Secondly, the exponential model is a plausible way to describe changes in the search intensity over time, as the Poisson coefficient in any week *w* is:

$$ln\left(\frac{y_{syw}^{trigg\quad /pre-Roe}}{y_{syw=-}^{trigger/pre-Roe}}\right) - ln\left(\frac{y_{syw}^{Other}}{y_{syw=-}^{Other}}\right)$$

and captures in our main model the differential percentage change in the number of searches, relative to the week before the leak, in trigger/pre-Roe abortion ban states compared to other states. Thus, a coefficient value of 0 in any week would indicate equal magnitude increases in search intensity, relative to the week prior to the leak, in trigger/pre-Roe abortion ban and other states following the May 2, 2022 leak. On the other hand, coefficient values progressively

© 2023 Gupta et al. *JAMA Health Forum.*

greater than 0 indicate larger relative increases in search intensity in trigger/pre-Roe abortion ban states than states that protect abortion access following the leak. Overall, our Poisson event study design with controls for secular changes, seasonality, and time invariant state differences, allows us to go beyond earlier literature by presenting estimates of the extent to which Dobbs ruling was associated with differential changes in internet based reproductive care related information seeking behaviors beyond baseline state differences. Poisson coefficients and 95%CIs are reported for the 10 weeks leading up to the leak and for the subsequent 9 weeks (Figures 3-4, and eFigures 1-8).

In our main analyses we capture the immediate impact of the Dobbs ruling by distinguishing between the U.S. states where access to reproductive health did not immediately change on June 24th, 2022, vs the thirteen states with 'trigger' laws that banned or curtail abortion immediately - Arkansas, Idaho, Kentucky, Louisiana, Mississippi, Missouri, North Dakota, Oklahoma, South Dakota, Tennessee, Texas, Utah and Wyoming, and four states with pre-*Roe* abortion bans on the books that became enforceable again - Arizona, Michigan, Wisconsin and West Virginia - following the U.S. Supreme Court's reversal of Roe vs Wade. However, there are growing concerns that Republican states without trigger/pre-Roe ban laws may also soon criminalize abortion due to their popular 'pro-life' sentiment. Therefore, in secondary analyses we estimate similar event-study models comparing changes in internet searches for abortion and contraception terms in Republican 'red' states, relative to Democrat 'blue' states, defined as consistently having a Republican or Democrat Governor between January 2015-present (refer eFigure 3 for detailed classification). Finally, in further sensitivity analysis (eFigure 7) we also estimate an event study that traces out internet search responses in the ten weeks before and four weeks after the U.S. Supreme Court's formal repeal of the constitutional protection for abortion on June 24, 2022 (week before the ruling now serving as the reference week with a normalized value of 0).

© 2023 Gupta et al. *JAMA Health Forum.*

eTable 1. Mapping of states with laws that ban most or all abortions (trigger and pre-Roe ban states) and Democrat 'blue' and Republican 'red' states.

Notes: *Author's classification of states into Democrat ('blue') and Republican ('red') states based on whether the state consistently had either a Republican or a Democrat governor for the period of January 2015-July 2022. Eighteen states where the political affiliation of the governor switched during the study period were excluded from these analyses. Blue bold underline denotes the thirteen states with 'trigger' laws that banned or curtail abortion immediately - Arkansas, Idaho, Kentucky, Louisiana, Mississippi, Missouri, North Dakota, Oklahoma, South Dakota, Tennessee, Texas, Utah and Wyoming - following the apex court's reversal of Roe vs Wade. Yellow bold underline denotes the additional four states with pre-Roe abortion bans that became enforceable again - Arizona, Michigan, Wisconsin and West Virginia - following the Dobbs ruling.*

**Blue States**	**Red States**	**Swing States**
CA, CO, CT, DC, DE, MN, NY, OR, PA, RI, WA	AL, **AR**, **AZ**, FL, GA, IA, **ID**, IN, MA, MD, **MS**, **ND**, NE, OH, **OK**, SC, **SD**, **TN**, **TX**, **UT**, **WY**	AK, HI, KS, **KY**, **LA**, ME, **MI**, **MO**, MT, NC, NH, NJ, NM, NV, VA, VT, **WI**, **WV**

© 2023 Gupta et al. *JAMA Health Forum.*

eTable 2. Estimated number of searches for abortion and contraception terms in trigger/ pre-*Roe* abortion ban states and other states with laws protecting abortion access between January 2, 2022 and July 16, 2022.

Notes: *The table presents regression adjusted estimates of the expected counts of abortion and contraception terms searches. Refer to Material and Methods section for details on construction of abortion and contraception search terms. Refer to Figure 3 for the corresponding estimates of the differential percentage change in search frequency, i.e., differences in log expected counts of search relative to the week prior to the leak, in trigger/pre-Roe abortion ban states relative to other states.*

	Trigger/pre-Roe ban states		Other states	
Week starting on	Abortion terms	Contraception terms	Abortion terms	Contraception terms
2-Jan-22	16932	57511	18132	47595
9-Jan-22	16778	59578	17968	49307
16-Jan-22	18805	57860	20138	47885
23-Jan-22	19057	57735	20408	47781
30-Jan-22	17090	58883	18302	48731
6-Feb-22	16976	58570	18179	48472
13-Feb-22	18153	56864	19440	47060
20-Feb-22	14078	55944	19456	48289
27-Feb-22	13077	56522	19780	48580
6-Mar-22	15380	57482	20866	48436
13-Mar-22	16655	57527	20482	46700
20-Mar-22	16804	59849	19588	47959
27-Mar-22	15868	54265	20203	47877
3-Apr-22	18715	55143	21643	47099
10-Apr-22	17935	60134	21098	49644
17-Apr-22	16201	60162	19727	49389
24-Apr-22	16302	56056	20023	49669
1-May-22	75746	64808	61426	51275
8-May-22	50246	64993	40455	50515
15-May-22	26645	61629	30245	48669
22-May-22	20545	60604	26288	49604
29-May-22	15603	59587	23465	48027
5-Jun-22	14755	59433	20353	48026
12-Jun-22	16043	60258	20775	47708
19-Jun-22	150602	72706	100183	52722
26-Jun-22	146720	82134	93141	54493
3-Jul-22	51330	65802	40460	49715
10-Jul-22	32197	63246	30568	49663

© 2023 Gupta et al. *JAMA Health Forum.*

eTable 3. Estimated number of searches for abortion and contraception terms in non- trigger/pre-Roe ban “red” states vs “blue” states between January 2, 2022 and July 16, 2022.

Notes: *The table presents regression adjusted estimates of the expected counts of abortion and contraception terms searches. States stratified by Governor’s political affiliation - consistently Democrat “blue” states (ten “blue” states - CA, CO, CT, DE, MN, NY, OR, PA, RI, WA - and Washington DC have consistently maintained a Democrat Governor between 2015-2022) as compared with consistently Republican “red” states (21 “red” states - AL, AR, AZ, FL, GA, IA, ID, IN, MA, MD, MS, ND, NE, OH, OK, SC, SD, TN, TX, UT, WY - with a Republican Governor between 2015-2022). For these analyses we excluded the eighteen states with transitions in political affiliations in gubernatorial office between 2015-2022 (AK, HI, KS, KY, LA, ME, MI, MO, MT, NC, NH, NJ, NM, NV, VA, VT, WI, WV). Refer to SI Table B.1 for details including mapping of state political affiliation with trigger/pre-Roe ban status. Refer to Material and Methods section for details on construction of abortion and contraception search terms. Refer to Figure 4 for the corresponding estimates of the differential percentage change in search frequency, i.e., differences in log expected counts of search relative to the week prior to the leak, in non-trigger/pre-Roe ban “red” states relative to “blue” state.*

	"Red" non-trigger/pre-Roe states states		"Blue"non-trigger/pre-Roe states states	
Week starting on	Abortion terms	Contraception terms	Abortion terms	Contraception terms
2-Jan-22	17523	52104	19243	44001
9-Jan-22	16907	53640	18567	45299
16-Jan-22	18424	51877	20233	43810
23-Jan-22	19227	51527	21115	43515
30-Jan-22	17021	52921	18692	44692
6-Feb-22	16870	52250	18527	44125
13-Feb-22	17940	51631	19702	43602
20-Feb-22	13284	50928	20766	44808
27-Feb-22	14006	50791	20522	45274
6-Mar-22	14255	51273	21896	44822
13-Mar-22	15940	50019	22123	42929
20-Mar-22	15299	52144	20671	44660
27-Mar-22	16128	49926	20720	43793
3-Apr-22	18211	49645	22533	43384
10-Apr-22	17188	52640	22864	46186
17-Apr-22	16473	53766	20895	45567
24-Apr-22	15791	53020	20881	45777
1-May-22	73587	57227	62560	47051
8-May-22	46801	56779	42983	46124
15-May-22	24582	53423	31970	44860
22-May-22	20274	51936	27889	45029
29-May-22	18053	50952	24542	43206
5-Jun-22	17906	54258	20969	43070
12-Jun-22	15940	52361	22274	43766
19-Jun-22	141491	65609	94568	48005
26-Jun-22	133878	70328	86841	48421
3-Jul-22	50313	57393	40174	44975
10-Jul-22	31674	55920	31151	45737

© 2023 Gupta et al. *JAMA Health Forum.*

eTable 4. Estimated number of searches for specific abortion terms in trigger/ pre-*Roe* abortion ban states and other states with laws protecting abortion access between January 2, 2022 and July 16, 2022.

Notes: *The table presents regression adjusted estimates of the expected counts searches of specific abortion terms. Refer to SI eFigure 1 for the corresponding estimates of the differential percentage change in search frequency, i.e., differences in log expected counts of search relative to the week prior to the leak, in trigger/pre-Roe abortion ban states relative to other states.*

	Trigger/pre-Roe ban states					Other States				
Week starting on	Abortion	Abortion Pill	Plan B	Mifepristone	Misoprostol	Abortion	Abortion Pill	Plan B	Mifepristone	Misoprostol
2-Jan-22	9529	834	5250	142	382	10539	922	5193	141	351
9-Jan-22	9622	885	4922	221	410	10643	979	4869	218	377
16-Jan-22	11821	764	5088	135	403	13075	845	5033	134	371
23-Jan-22	12578	853	4528	144	515	13912	943	4479	143	474
30-Jan-22	10067	809	4857	197	491	11135	895	4805	196	452
6-Feb-22	9827	864	4968	135	459	10869	955	4915	134	422
13-Feb-22	10047	835	5718	150	518	11113	924	5656	149	476
20-Feb-22	7821	825	4834	139	476	12214	924	4829	145	428
27-Feb-22	7631	877	4021	201	371	12713	864	4814	113	434
6-Mar-22	8789	991	5008	163	449	12780	995	5359	155	479
13-Mar-22	10292	789	4888	72	622	12931	929	5013	109	552
20-Mar-22	10276	825	5172	102	440	11818	834	5213	169	449
27-Mar-22	9697	821	4827	148	393	12883	904	4953	162	419
3-Apr-22	12750	776	4488	151	567	14347	927	5016	175	429
10-Apr-22	12448	677	4268	161	401	14085	996	4650	176	519
17-Apr-22	9684	628	5399	75	425	12288	868	5036	152	409
24-Apr-22	9492	943	5237	171	479	12706	913	4893	152	465
1-May-22	65353	1638	7541	460	807	57379	1347	5601	236	648
8-May-22	36102	1031	12342	148	641	33371	989	6318	187	464
15-May-22	19067	860	5906	206	631	23880	1039	4998	147	489
22-May-22	13939	759	5383	165	318	18727	865	5553	169	499
29-May-22	8756	681	5593	101	484	15157	909	5824	132	453
5-Jun-22	8775	760	4789	157	293	12738	962	5117	142	416
12-Jun-22	9824	819	4918	169	333	12995	866	5327	141	428
19-Jun-22	136684	2166	10546	520	747	98777	1559	6831	453	569
26-Jun-22	129970	2403	12874	665	887	89469	1623	7542	439	670
3-Jul-22	41796	1732	7102	206	518	34201	1197	5687	148	423
10-Jul-22	24397	981	6077	278	496	23944	944	5345	131	420

© 2023 Gupta et al. *JAMA Health Forum.*

eTable 5. Estimated number of searches for specific contraception terms in trigger/ pre-*Roe* abortion ban states and other states with laws protecting abortion access between January 2, 2022 and July 16, 2022.

Notes: *The table presents regression adjusted estimates of the expected counts searches of specific contraception terms. Refer to SI eFigure 2 for the corresponding estimates of the differential percentage change in search frequency, i.e., differences in log expected counts of search relative to the week prior to the leak, in trigger/pre-Roe abortion ban states relative to other states.*

	**Trigger/pre-Roe ban states**			**Other States**		
Week starting on	Contraceptive pills/ condoms	Implants/IUDs	Tubal Ligation/ Vasectomy	Contraceptive pills/ condoms	Implants/ IUDs	Tubal Ligation/ Vasectomy
2-Jan-22	42382	11299	3199	33316	10859	2706
9-Jan-22	43050	12182	3487	33841	11707	2951
16-Jan-22	42410	11299	3455	33338	10859	2924
23-Jan-22	41523	11736	3708	32641	11279	3138
30-Jan-22	42086	12294	3622	33083	11815	3065
6-Feb-22	42196	11930	3494	33169	11465	2957
13-Feb-22	41289	11462	3297	32456	11015	2790
20-Feb-22	40271	11668	3212	33200	11159	3167
27-Feb-22	42317	10462	3010	33488	11358	3010
6-Mar-22	41380	11930	3369	33224	11253	3191
13-Mar-22	43134	10409	3148	32052	10846	2967
20-Mar-22	43284	11987	3926	33222	10964	3035
27-Mar-22	39727	10702	3230	32982	10855	3229
3-Apr-22	40369	10455	3386	32494	10698	3186
10-Apr-22	44809	11390	3093	34516	10906	3314
17-Apr-22	43970	12028	3488	34269	11242	3038
24-Apr-22	41304	11164	3033	34408	11397	3105
1-May-22	44019	13431	6385	34252	11991	4322
8-May-22	43990	14795	5025	34440	11666	3683
15-May-22	45388	11597	4034	33816	10913	3249
22-May-22	45109	11327	3685	34906	10705	3207
29-May-22	45133	10242	3627	34290	10288	2719
5-Jun-22	44118	10960	3601	33425	10728	3105
12-Jun-22	44698	10921	3893	33186	10767	3130
19-Jun-22	48381	13061	8824	35015	11973	4637
26-Jun-22	50613	15568	13666	34892	12208	6622
3-Jul-22	46709	12078	6138	34444	10667	3865
10-Jul-22	45551	12077	4748	34012	11164	3721

© 2023 Gupta et al. *JAMA Health Forum.*

eTable 6. Estimated number of searches for abortion and contraception terms in "red" versus "blue" states between January 2, 2022 and July 16, 2022.

Notes: *The table presents regression adjusted estimates of the expected counts of abortion and contraception terms searches. Refer to SI Table B.1 for details including mapping of state political affiliation with trigger/pre-Roe ban status. 10 Red states (i.e. with Republican Governor from 2015 through to 2022)- AL, FL, GA, IA, IN, MA, MD, NE, OH and SC - did not have existing 'trigger' and pre-Roe ban laws, that would immediately criminalize abortion following the Dobbs ruling. However, there are growing concerns that these states may soon adopt abortion criminalization legislation as well. There are no consistent (i.e. Democrat Governor from 2015 through to 2022) Blue 'trigger' and pre-Roe ban states. Refer to Material and Methods section for details on construction of abortion and contraception search terms. Refer to eFigure 3 for the corresponding estimates of the differential percentage change in search frequency, i.e., differences in log expected counts of search relative to the week prior to the leak, in "red" states relative to "blue" state.*

	**"Red" states**		**"Blue" states**	
Week starting on	Abortion terms	Contraception terms	Abortion terms	Contraception terms
2-Jan-22	17006	54024	19229	43818
9-Jan-22	16995	55653	19216	45139
16-Jan-22	18700	53802	21145	43637
23-Jan-22	18962	54005	21441	43802
30-Jan-22	16972	54873	19191	44506
6-Feb-22	16776	54706	18969	44371
13-Feb-22	17777	53087	20101	43058
20-Feb-22	14275	51918	21977	45325
27-Feb-22	13582	52353	21548	44900
6-Mar-22	14855	53516	23842	44923
13-Mar-22	16315	52617	22849	42610
20-Mar-22	16295	55491	21633	44038
27-Mar-22	16017	50545	22242	44293
3-Apr-22	18777	51715	23901	43658
10-Apr-22	17599	55032	23360	45668
17-Apr-22	16420	56379	21773	45312
24-Apr-22	16154	54057	22018	45596
1-May-22	73507	59708	55204	46724
8-May-22	47066	59740	39012	46081
15-May-22	25847	56787	32482	45398
22-May-22	20685	55095	28411	46031
29-May-22	16651	54509	25761	42950
5-Jun-22	16155	55465	21789	42862
12-Jun-22	16307	56024	23109	44029
19-Jun-22	143182	68235	81041	47755
26-Jun-22	139434	75505	74765	47054
3-Jul-22	51474	60671	36871	45534
10-Jul-22	31997	58801	29470	45549

© 2023 Gupta et al. *JAMA Health Forum.*

eTable 7. Estimated number of searches for abortion and contraception terms in immediately impacted trigger states and other states with laws protecting abortion access between January 2, 2022 and July 16, 2022.

Notes: *The table presents regression adjusted estimates of the expected counts of abortion and contraception terms searches in trigger ban states relative to other states. Pre-Roe ban states are excluded from the analysis. Refer to Figure 1 for details of state laws – trigger and pre-Roe abortion bans - restricting abortion access. Refer to Material and Methods section for details on construction of abortion and contraception search terms. Refer to SI eFigure 4 for the corresponding estimates of the differential percentage change in search frequency relative to the week prior to the leak, in trigger ban states relative to other states.*

	**Trigger states**		**Other states**	
Week starting on	Abortion terms	Contraception terms	Abortion terms	Contraception terms
2-Jan-22	17002	59109	17975	47882
9-Jan-22	16990	61034	17963	49441
16-Jan-22	19156	59181	20253	47940
23-Jan-22	19338	58932	20445	47739
30-Jan-22	17276	60257	18266	48812
6-Feb-22	17253	59862	18241	48492
13-Feb-22	18460	58019	19517	46999
20-Feb-22	14688	56800	19089	48109
27-Feb-22	13287	56955	19402	48645
6-Mar-22	15674	58854	20615	48343
13-Mar-22	16729	58127	20169	46775
20-Mar-22	16982	61629	19470	47973
27-Mar-22	15826	54674	20048	47900
3-Apr-22	19292	55912	21388	47027
10-Apr-22	18634	60364	20935	49704
17-Apr-22	16943	61997	19515	49344
24-Apr-22	16601	57701	19697	49654
1-May-22	74087	65735	62504	51398
8-May-22	50926	66622	40984	50563
15-May-22	27182	62710	30193	48629
22-May-22	21099	61314	26121	49648
29-May-22	16125	61187	23148	47899
5-Jun-22	14736	59763	20144	47958
12-Jun-22	16244	62345	20561	47824
19-Jun-22	145340	73304	102761	52995
26-Jun-22	144513	83301	95442	54732
3-Jul-22	50665	66431	41005	49859
10-Jul-22	32234	64610	30785	49773

© 2023 Gupta et al. *JAMA Health Forum.*

eTable 8. Estimated number of searches for abortion and contraception terms in pre-*Roe* abortion ban states and other states with laws protecting abortion access between January 2, 2022 and July 16, 2022.

Notes: *The table presents regression adjusted estimates of the expected counts of abortion and contraception terms searches in pre-Roe abortion ban states relative to other states. Trigger ban states are excluded from the analysis. Refer to Figure 1 for details of state laws – trigger and pre-Roe abortion bans - restricting abortion access. Refer to Material and Methods section for details on construction of abortion and contraception search terms. Refer to SI eFigure 5 for the corresponding estimates of the differential percentage change in search frequency relative to the week prior to the leak in pre-Roe abortion ban states relative to other states,*

	**Pre-*Roe* abortion ban states**		**Other states**	
Week starting on	Abortion terms	Contraception terms	Abortion terms	Contraception terms
2-Jan-22	16641	53569	18054	47629
9-Jan-22	16057	55826	17421	49636
16-Jan-22	17990	53880	19518	47906
23-Jan-22	18608	53503	20188	47571
30-Jan-22	16516	55121	17919	49010
6-Feb-22	16456	54162	17853	48157
13-Feb-22	17725	53429	19230	47505
20-Feb-22	12098	53164	18395	47896
27-Feb-22	12394	55116	18804	48452
6-Mar-22	14426	53022	19545	48224
13-Mar-22	16415	55577	19787	46918
20-Mar-22	16227	54065	18763	48352
27-Mar-22	16006	52936	19130	47663
3-Apr-22	16837	52646	20779	46845
10-Apr-22	15662	59386	20448	49808
17-Apr-22	13790	54198	18934	49437
24-Apr-22	15331	50707	19284	49658
1-May-22	81135	61797	65722	51579
8-May-22	48035	59696	42480	50763
15-May-22	24900	58113	29671	48275
22-May-22	18743	58296	25663	49129
29-May-22	13907	54389	22501	48107
5-Jun-22	14819	58362	19669	48223
12-Jun-22	15389	53474	19894	47540
19-Jun-22	167705	70760	108998	53114
26-Jun-22	153894	78340	101089	55823
3-Jul-22	53491	63757	42355	49499
10-Jul-22	32075	58812	31344	49489

© 2023 Gupta et al. *JAMA Health Forum.*

eTable 9. Estimated number of searches for abortion and contraception terms in Trigger/ pre-*Roe* abortion ban states, excluding early adopters Texas and Oklahoma, and other states with laws protecting abortion access between January 2, 2022 and July 16, 2022.

Notes: *The table presents regression adjusted estimates of the expected counts of abortion and contraception terms searches in Trigger/ pre-Roe abortion ban states relative to other states. Early abortion ban states of Texas and Oklahoma are excluded from the analysis. Refer to Material and Methods section for details on construction of abortion and contraception search terms. Refer to SI eFigure 6 for the corresponding estimates of the differential percentage change in search frequency relative to the week prior to the leak in Trigger/ pre-Roe abortion ban states excluding Texas and Oklahoma relative to other states,*

	**Trigger *pre-Roe* abortion ban states excl. TX and OK**		**Other states**	
Week starting on	Abortion terms	Contraception terms	Abortion terms	Contraception terms
2-Jan-22	16597	57688	18091	47578
9-Jan-22	16566	59828	18057	49343
16-Jan-22	18445	58121	20105	47935
23-Jan-22	18739	57908	20426	47759
30-Jan-22	16808	59120	18321	48759
6-Feb-22	16691	58876	18193	48558
13-Feb-22	17843	57254	19449	47220
20-Feb-22	14128	56529	19338	48252
27-Feb-22	12838	56529	19692	48612
6-Mar-22	15346	57746	20699	48347
13-Mar-22	16743	57716	20365	46718
20-Mar-22	16273	60265	19479	48001
27-Mar-22	15650	54247	20067	47888
3-Apr-22	17350	55434	21540	46888
10-Apr-22	16392	60173	21019	49658
17-Apr-22	15749	60139	19617	49417
24-Apr-22	15896	56286	19861	49712
1-May-22	76346	65213	62110	51276
8-May-22	50246	65370	40777	50517
15-May-22	26051	61972	29960	48617
22-May-22	19803	60696	26152	49540
29-May-22	15241	60105	23257	48010
5-Jun-22	14400	60003	20311	48065
12-Jun-22	15800	60923	20678	47687
19-Jun-22	154249	72996	101608	52765
26-Jun-22	148961	82807	94433	54732
3-Jul-22	51799	66405	40704	49736
10-Jul-22	32232	63316	30679	49718

© 2023 Gupta et al. *JAMA Health Forum.*

eTable 10. Estimated number of searches for abortion and contraception terms in early adopters Texas and Oklahoma, and other states with laws protecting abortion access between January 2, 2022 and July 16, 2022.

Notes: *The table presents regression adjusted estimates of the expected counts of abortion and contraception terms searches in early abortion ban states of Texas and Oklahoma relative to other states. Other non- trigger/pre-Roe abortion ban are excluded from the analysis. Refer to Material and Methods section for details on construction of abortion and contraception search terms. Refer to SI eFigure 7 for the corresponding estimates of the differential percentage change in search frequency relative to the week prior to the leak in Texas and Oklahoma relative to other non- trigger/pre-Roe abortion ban states,*

	**TX and OK only**		**Other States**	
Week starting on	Abortion terms	Contraception terms	Abortion terms	Contraception terms
2-Jan-22	19556	56603	17911	48050
9-Jan-22	18865	58651	17279	49788
16-Jan-22	21500	56438	19692	47910
23-Jan-22	22067	55991	20212	47530
30-Jan-22	19475	57835	17838	49096
6-Feb-22	19552	56593	17908	48041
13-Feb-22	21090	55621	19317	47216
20-Feb-22	13705	51563	18102	47711
27-Feb-22	14867	56477	18467	48482
6-Mar-22	15637	55495	19429	48212
13-Mar-22	15995	56104	19557	46988
20-Mar-22	20787	56732	18736	48324
27-Mar-22	17504	54399	19089	47666
3-Apr-22	28946	52967	20594	47004
10-Apr-22	29509	59839	20341	49863
17-Apr-22	19591	60334	18805	49348
24-Apr-22	19348	54330	19089	49583
1-May-22	71248	61774	66188	51728
8-May-22	50244	62158	42719	50820
15-May-22	31103	59054	29913	48275
22-May-22	26110	59912	25613	49242
29-May-22	18318	55705	22362	47975
5-Jun-22	17418	55161	19469	48097
12-Jun-22	17859	55267	19748	47695
19-Jun-22	123251	70532	110341	53393
26-Jun-22	129913	77085	102266	55852
3-Jul-22	47814	61279	42696	49633
10-Jul-22	31935	62719	31460	49543

© 2023 Gupta et al. *JAMA Health Forum.*

eTable 11. Estimated number of searches for abortion and contraception terms in trigger/ pre-*Roe* abortion ban states and other states with laws protecting abortion access between January 2, 2022 and July 16, 2022.

Notes: *The table presents regression adjusted estimates of the expected counts of abortion and contraception terms searches. Refer to Material and Methods section for details on construction of abortion and contraception search terms. Refer to SI eFigure 8 for the corresponding estimates of the differential percentage change in search frequency, i.e., differences in log expected counts of search relative to the week prior to the ruling, in trigger/pre-Roe abortion ban states relative to other states.*

	Trigger/pre-Roe ban states		Other states	
Week starting on	Abortion terms	Contraception terms	Abortion terms	Contraception terms
2-Jan-22	16566	57357	18193	47582
9-Jan-22	16416	59419	18028	49293
16-Jan-22	18399	57705	20205	47872
23-Jan-22	18645	57580	20476	47768
30-Jan-22	16721	58725	18363	48718
6-Feb-22	16609	58413	18240	48459
13-Feb-22	17761	56711	19505	47047
20-Feb-22	16889	57723	18547	47886
27-Feb-22	16902	58117	18562	48214
6-Mar-22	18174	58165	19958	48253
13-Mar-22	18178	56492	19962	46865
20-Mar-22	17574	58166	19299	48254
27-Mar-22	17808	56995	19557	47282
3-Apr-22	19450	56413	21359	46799
10-Apr-22	17935	60134	21039	49598
17-Apr-22	16201	60162	19672	49344
24-Apr-22	16302	56056	19967	49623
1-May-22	75746	64808	61254	51228
8-May-22	50246	64993	40342	50468
15-May-22	26645	61629	30160	48624
22-May-22	20545	60604	26215	49558
29-May-22	15603	59587	23399	47983
5-Jun-22	14755	59433	20296	47982
12-Jun-22	16043	60258	20717	47664
19-Jun-22	150602	72706	99903	52673
26-Jun-22	146720	82134	92880	54442
3-Jul-22	51330	65802	40347	49669
10-Jul-22	32197	63246	30483	49618

© 2023 Gupta et al. *JAMA Health Forum.*

eTable 12. Estimated number of searches for "Planned Parenthood" in trigger/ pre-*Roe* abortion ban states and other states with laws protecting abortion access between January 2, 2022 and July 16, 2022.

Notes: *The table presents regression adjusted estimates of the number of "Planned Parenthood" searches. Refer to SI eFigure 9 for the corresponding estimates of the differential percentage change in search frequency, i.e., differences in log expected counts of search relative to the week prior to the leak, in trigger/pre-Roe abortion ban states relative to other states.*

Week starting on	**Trigger/pre-*Roe* ban states**	**Other States**
2-Jan-22	3264	4715
9-Jan-22	3302	4771
16-Jan-22	3270	4725
23-Jan-22	3300	4768
30-Jan-22	3056	4415
6-Feb-22	3301	4770
13-Feb-22	3161	4567
20-Feb-22	2287	4770
27-Feb-22	2623	4602
6-Mar-22	2791	4609
13-Mar-22	2673	4500
20-Mar-22	3005	4495
27-Mar-22	2496	4236
3-Apr-22	2591	4575
10-Apr-22	2619	4309
17-Apr-22	2650	4372
24-Apr-22	2685	4410
1-May-22	6470	6644
8-May-22	4934	5767
15-May-22	3284	5033
22-May-22	3321	4663
29-May-22	2619	4175
5-Jun-22	2865	4595
12-Jun-22	3238	4555
19-Jun-22	9112	8207
26-Jun-22	9088	7758
3-Jul-22	3985	5057
10-Jul-22	3008	5011

© 2023 Gupta et al. *JAMA Health Forum.*

eFigure 1. Abortion search term details.

Notes: As in the main analyses, the week prior to the treatment (leak) is set as a reference (coefficient value normalized to 0): light navy vertical bar. The week prior to the official ruling on June 24, 2022 is depicted using the darker navy vertical bar. In brown are the estimated coefficients (95% CI, gray band) in the Poisson model (differences in log expected counts of search relative to the reference week). The average search frequency of each term per state per day is provided at the bottom of each panel.

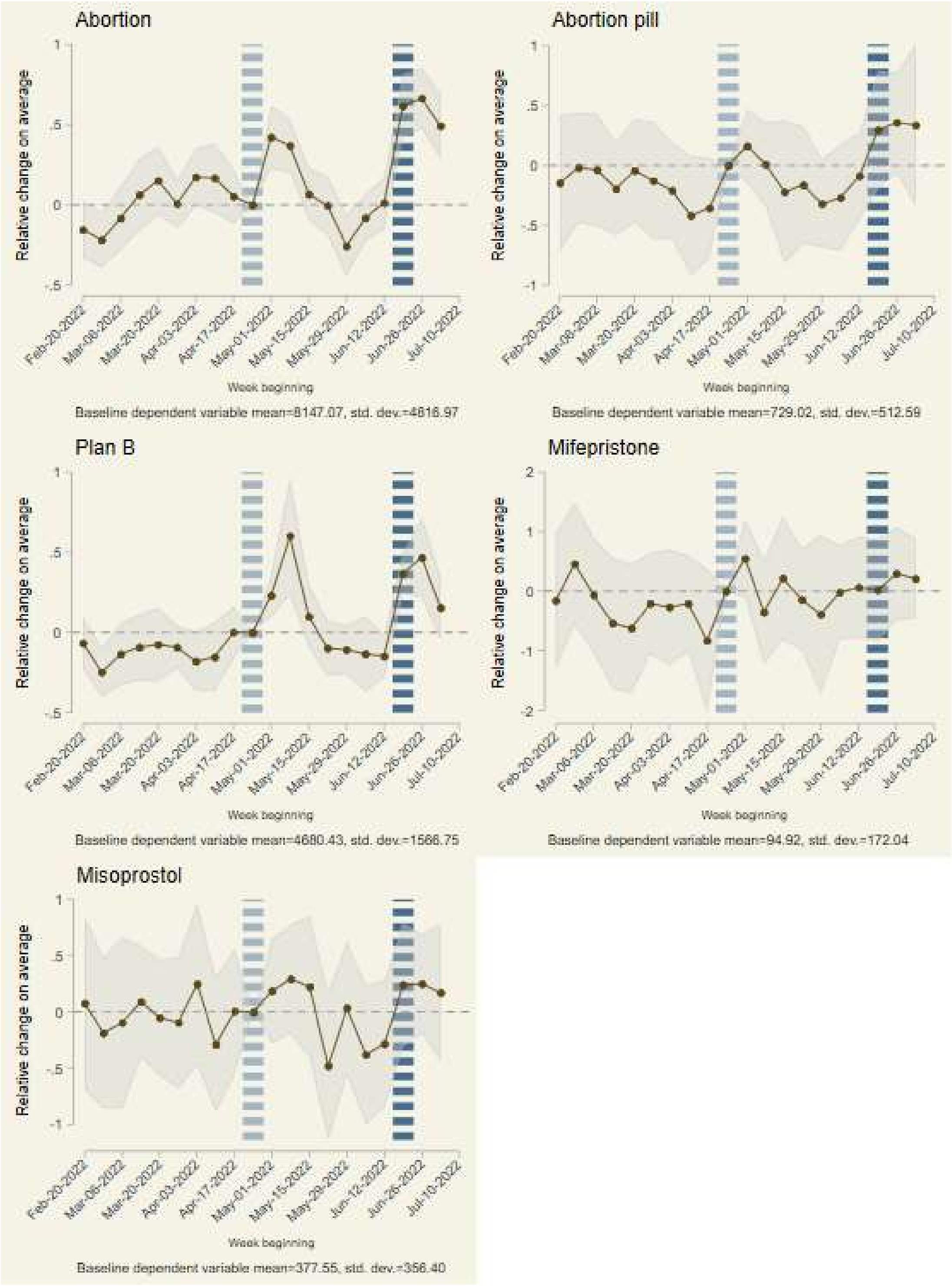

© 2023 Gupta et al. *JAMA Health Forum.*

eFigure 2. Contraception term details - Queries for short-acting, long-acting and permanent forms of contraception.

Notes: As in the main analyses, the week prior to the treatment (leak) is set as a reference (coefficient value normalized to 0): light navy vertical bar. The week prior to the official ruling on June 24, 2022 is depicted using the darker navy vertical bar. In brown are the estimated coefficients (95% CI, gray band) in the Poisson model (differences in log expected counts of search relative to the reference week). The average search frequency of each term per state per day is provided at the bottom of each panel.

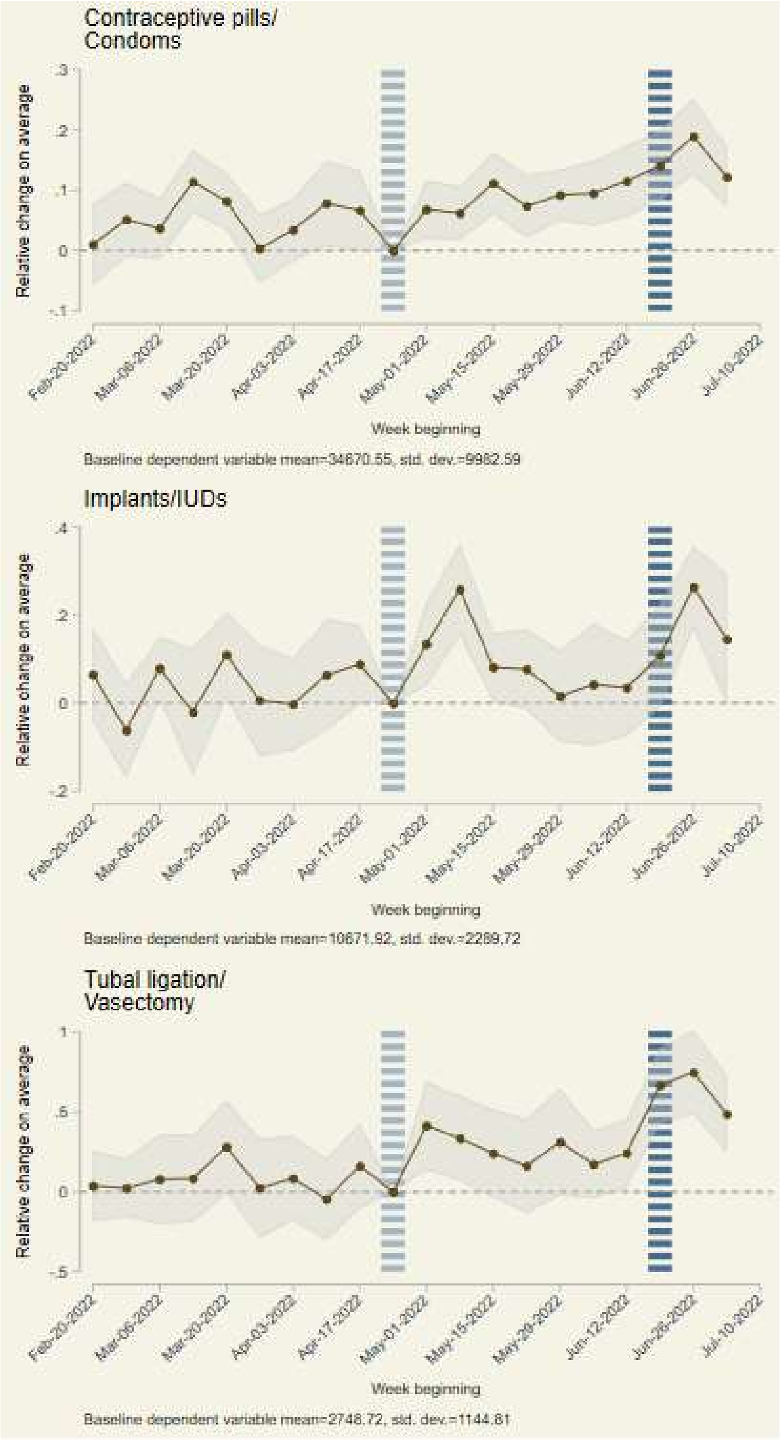

© 2023 Gupta et al. *JAMA Health Forum.*

eFigure 3. Time-varying effects of the leak of the U.S. Supreme Court's draft majority opinion in Red states compared to Blue states.

Notes: *10 Red states (i.e. with Republican Governor from 2015 through to 2022)- AL, FL, GA, IA, IN, MA, MD, NE, OH and SC - did not have existing 'trigger' and pre-Roe ban laws, that would immediately criminalize abortion following the Dobbs ruling. However, there are growing concerns that these states may soon adopt abortion criminalization legislation as well. There are no consistent (i.e. Democrat Governor from 2015 through to 2022) Blue 'trigger' and pre-Roe ban states. As in the main analyses, the week prior to the treatment (leak) is set as a reference (coefficient value normalized to 0): light navy vertical bar. The week prior to the official ruling on June 24, 2022 is depicted using the darker navy vertical bar. In brown are the estimated coefficients (95% CI, gray band) in the Poisson model (differences in log expected counts of search relative to the reference week). Refer to Material and Methods section for details on construction of abortion and contraception search terms. The average search frequency of each term per state per day is provided at the bottom of each panel.*

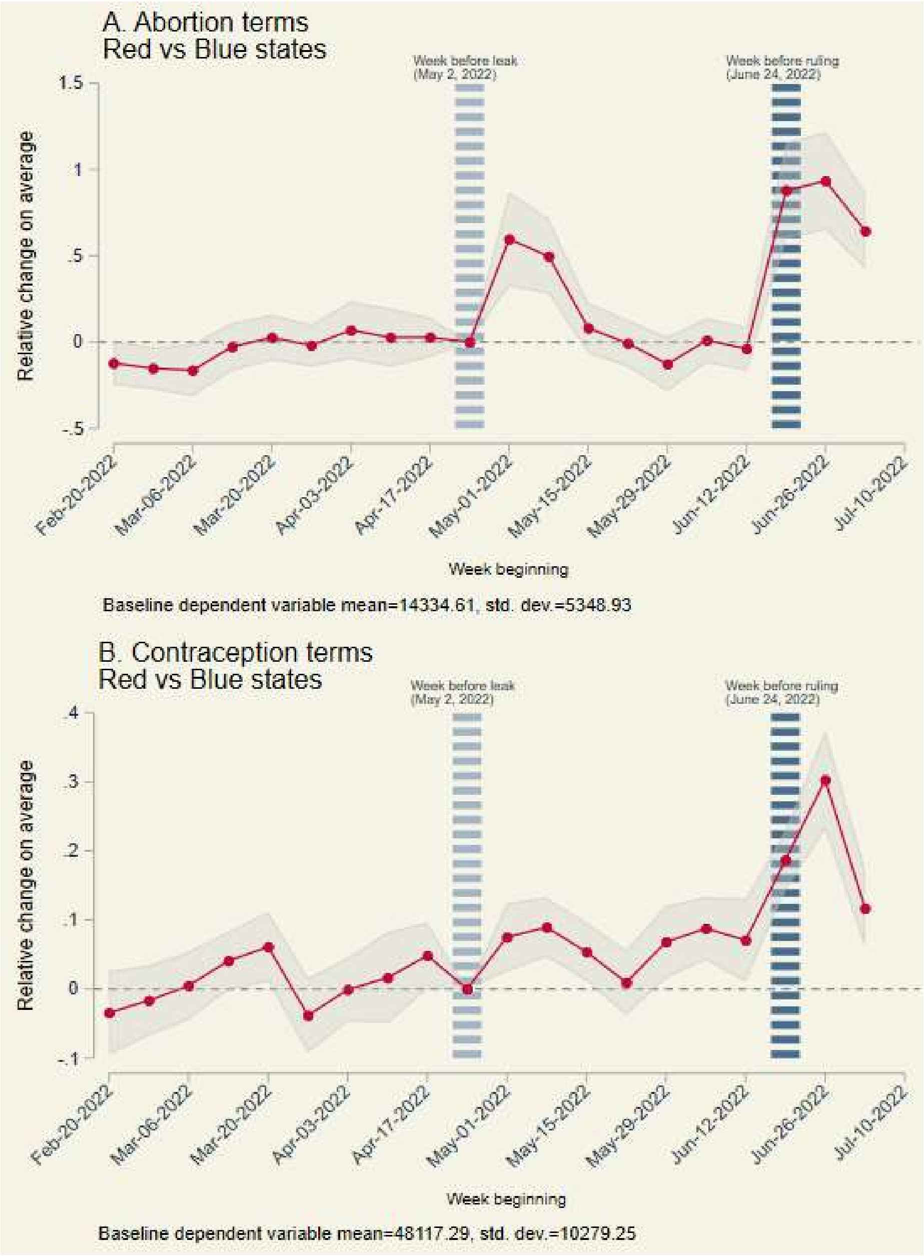

© 2023 Gupta et al. *JAMA Health Forum.*

eFigure 4. Time-varying effects of the leak of the U.S. Supreme Court's draft majority opinion in Dobbs on May 2, 2022, in trigger states vs States where state law protects abortion access. Treated sample includes only the thirteen 'trigger' states; pre-Roe ban states are excluded from the analysis.

Notes: As in the main analyses, the week prior to the treatment (leak) is set as a reference (coefficient value normalized to 0): light navy vertical bar. The week prior to the official ruling on June 24, 2022 is depicted using the darker navy vertical bar. In brown are the estimated coefficients (95% CI, gray band) in the Poisson model (differences in log expected counts of search relative to the reference week). Refer to Material and Methods section for details on construction of abortion and contraception search terms. The average search frequency of each term per state per day is provided at the bottom of each panel. Refer Figure 1 for list of the included thirteen 'trigger' states.

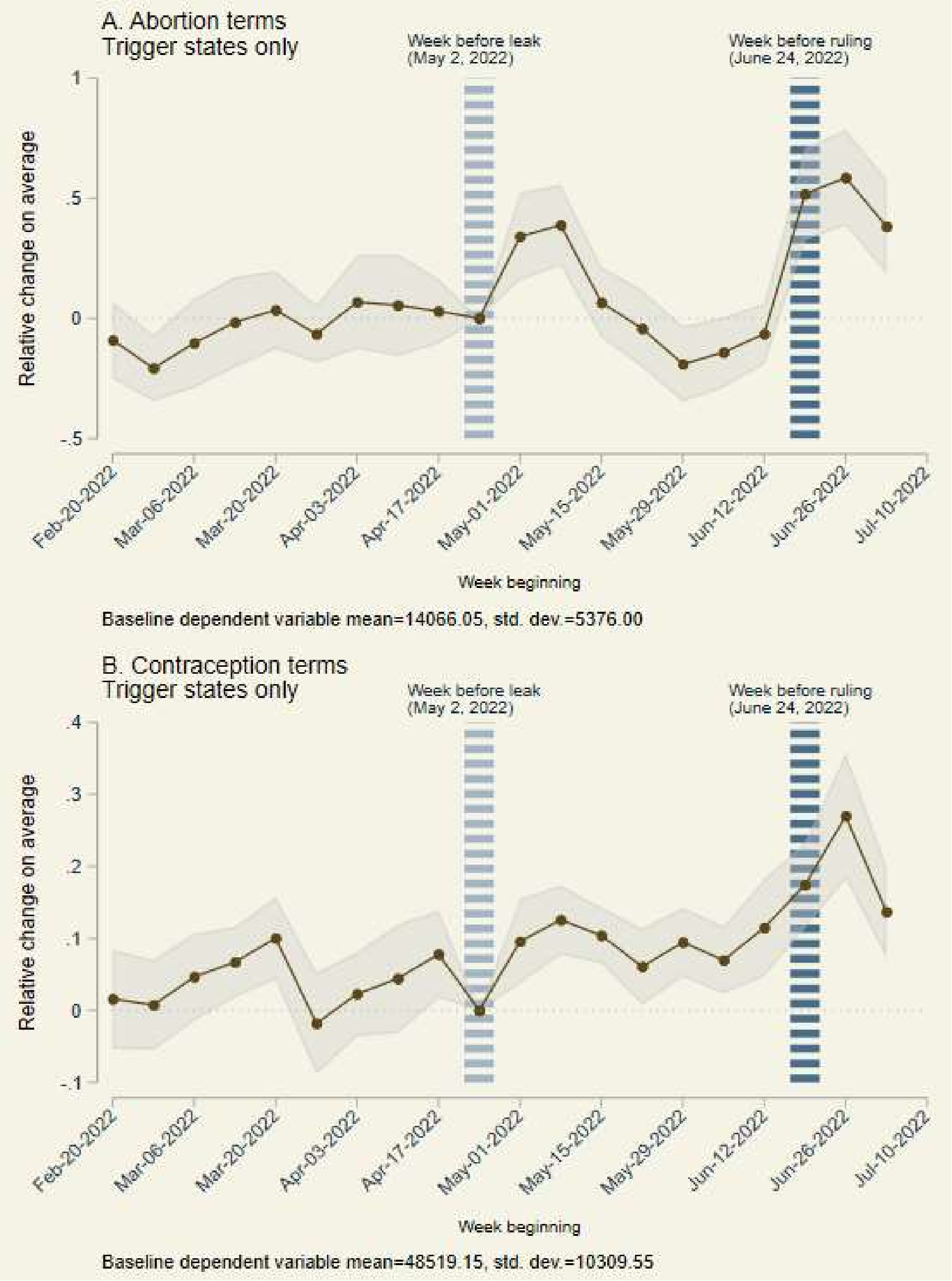

© 2023 Gupta et al. *JAMA Health Forum.*

eFigure 5. Time-varying effects of the leak of the U.S. Supreme Court's draft majority opinion in Dobbs v Jackson Women's Health Organization on May 2, 2022, in pre-Roe states vs States where state law protects abortion access. Treated sample includes only the four 'pre-Roe' states; 'trigger' states are excluded from the analysis.

Notes: As in the main analyses, the week prior to the treatment (leak) is set as a reference (coefficient value normalized to 0): light navy vertical bar. The week prior to the official ruling on June 24, 2022 is depicted using the darker navy vertical bar. In brown are the estimated coefficients (95% CI, gray band) in the Poisson model (differences in log expected counts of search relative to the reference week). Refer to Material and Methods section for details on construction of abortion and contraception search terms. The average search frequency of each term per state per day is provided at the bottom of each panel. Refer Figure 1 for list of the included four pre-Roe abortion ban states.

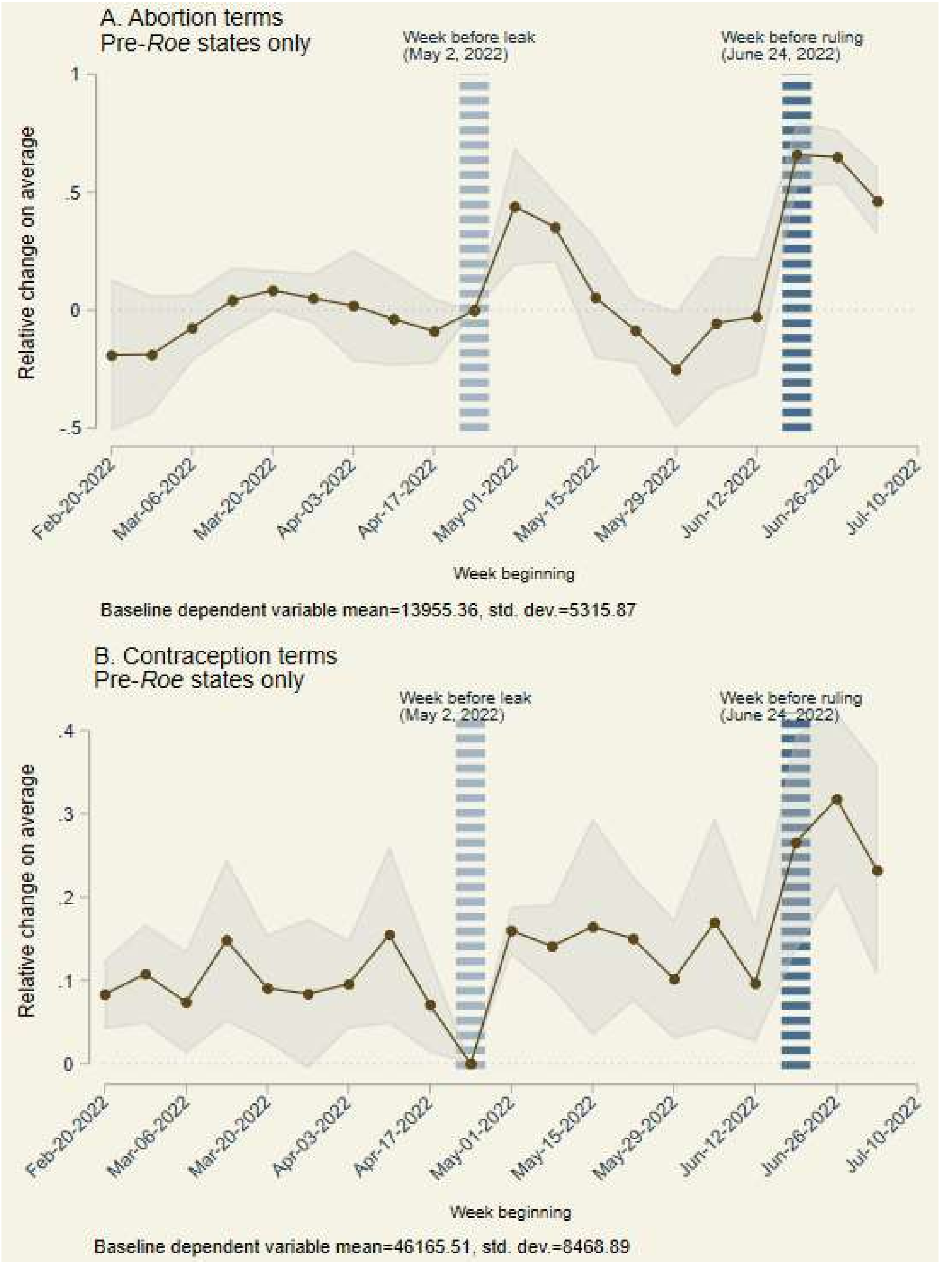

© 2023 Gupta et al. *JAMA Health Forum.*

eFigure 6. Time-varying effects of the leak of the U.S. Supreme Court's draft majority opinion in Dobbs on May 2, 2022, in trigger/pre-Roe ban states, excluding early adopters Texas and Oklahoma, vs States where state law protects abortion access.

Notes: As in the main analyses, the week prior to the treatment (leak) is set as a reference (coefficient value normalized to 0): light navy vertical bar. The week prior to the official ruling on June 24, 2022 is depicted using the darker navy vertical bar. In brown are the estimated coefficients (95% CI, gray band) in the Poisson model (differences in log expected counts of search relative to the reference week). Refer to Material and Methods section for details on construction of abortion and contraception search terms. The average search frequency of each term per state per day is provided at the bottom of each panel. Treated sample includes trigger and pre-Roe ban states (refer Figure 1 for list), excluding early adopters Texas and Oklahoma.

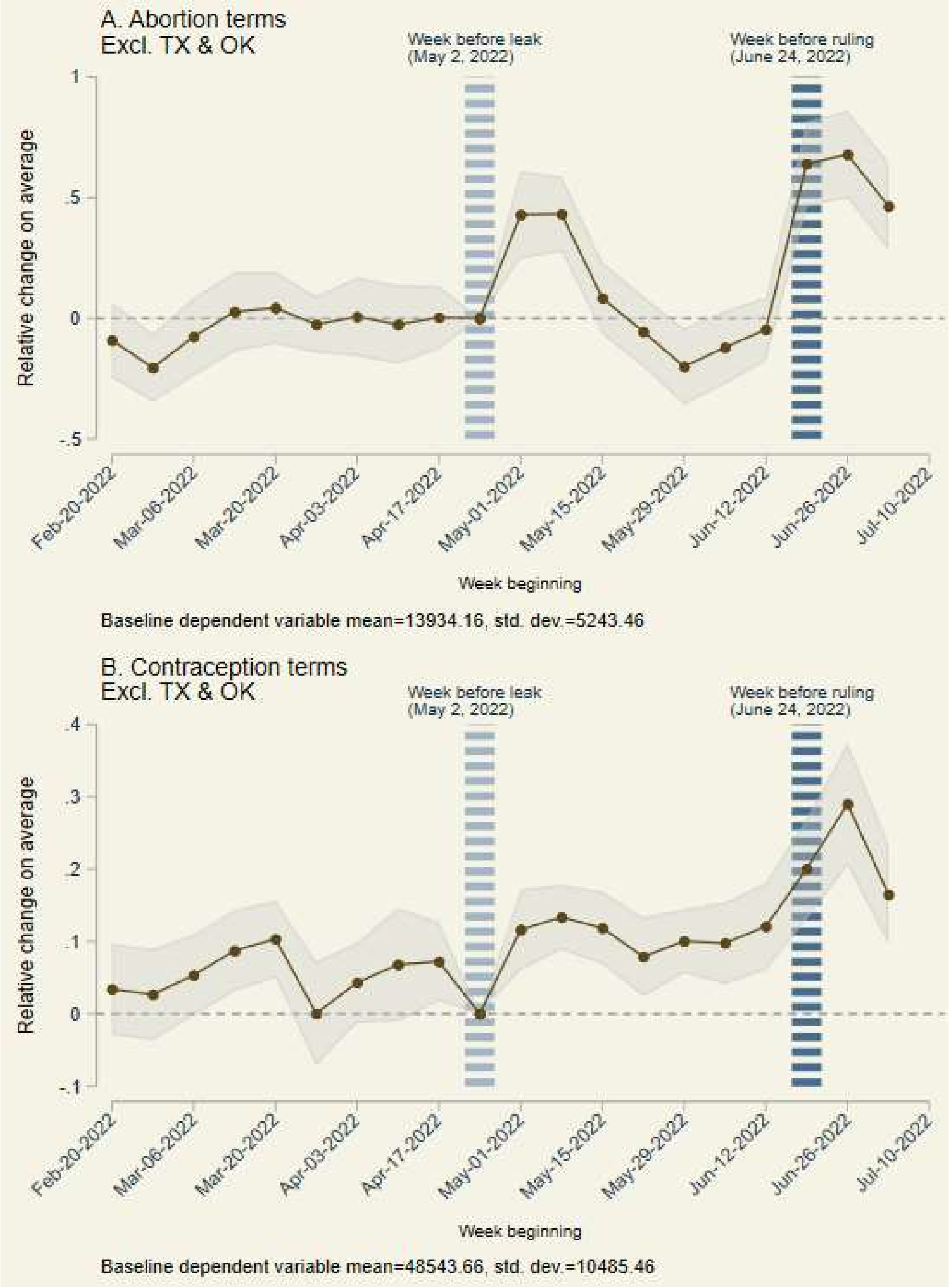

© 2023 Gupta et al. *JAMA Health Forum.*

eFigure 7. Time-varying effects of the leak of the U.S. Supreme Court's draft majority opinion in Dobbs on May 2, 2022, in early adopters Texas and Oklahoma, vs States where state law protects abortion access.

Notes: As in the main analyses, the week prior to the treatment (leak) is set as a reference (coefficient value normalized to 0): light navy vertical bar. The week prior to the official ruling on June 24, 2022 is depicted using the darker navy vertical bar. In brown are the estimated coefficients (95% CI, gray band) in the Poisson model (differences in log expected counts of search relative to the reference week). Refer to Material and Methods section for details on construction of abortion and contraception search terms. The average search frequency of each term per state per day is provided at the bottom of each panel. Treated sample includes <u>early adopters Texas and Oklahoma.</u>

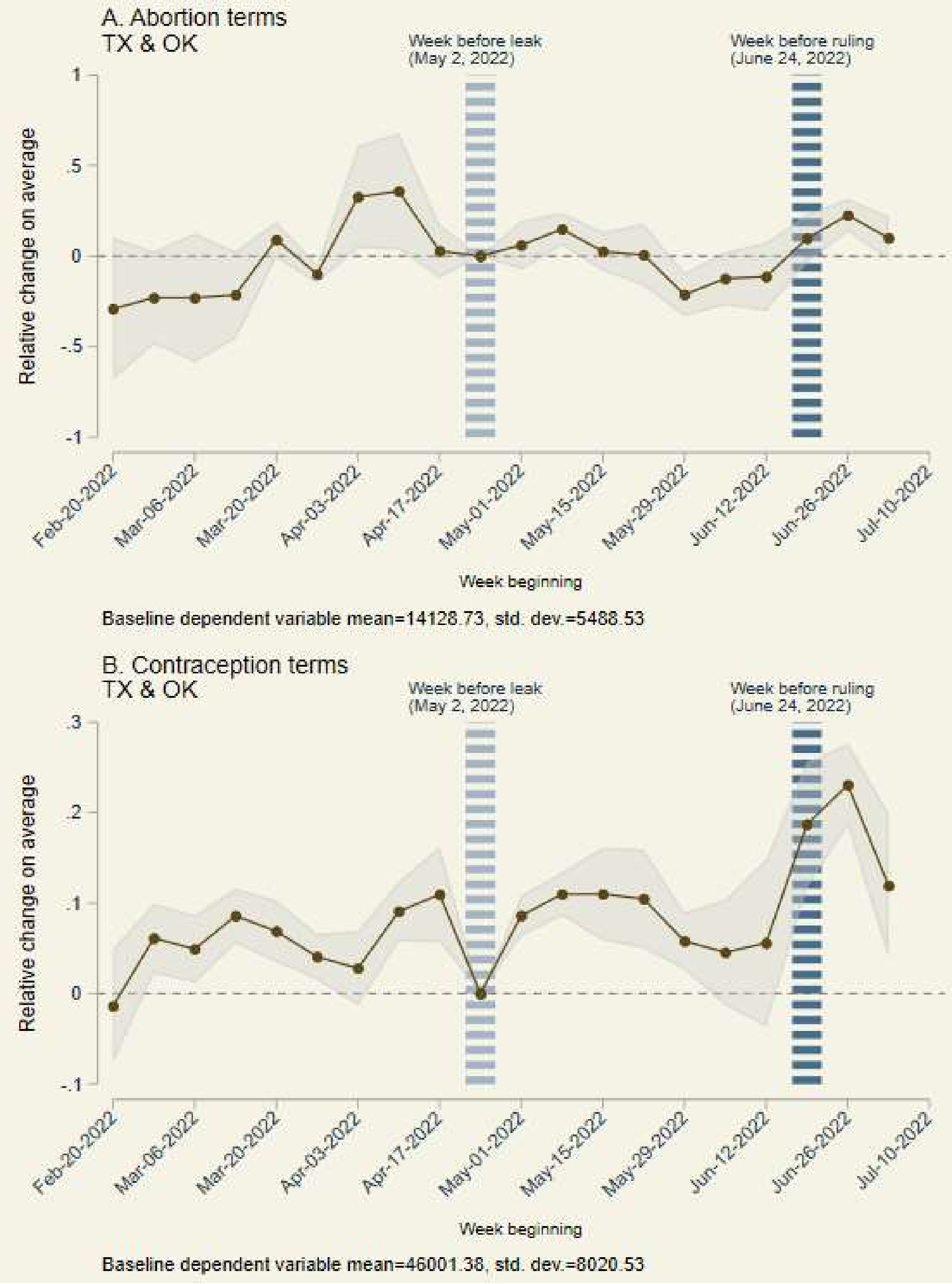

© 2023 Gupta et al. *JAMA Health Forum.*

eFigure 8. Time-varying effects of the U.S. Supreme Court's ruling in Dobbs v Jackson Women's Health Organization on June 24, 2022, in trigger and pre-Roe states vs States where state law protects abortion access.

Notes: *Treated sample includes both 'trigger' and 'pre-Roe ban' states. Reference period is the week before the one including June 24, 2022 (normalized value of 0).*

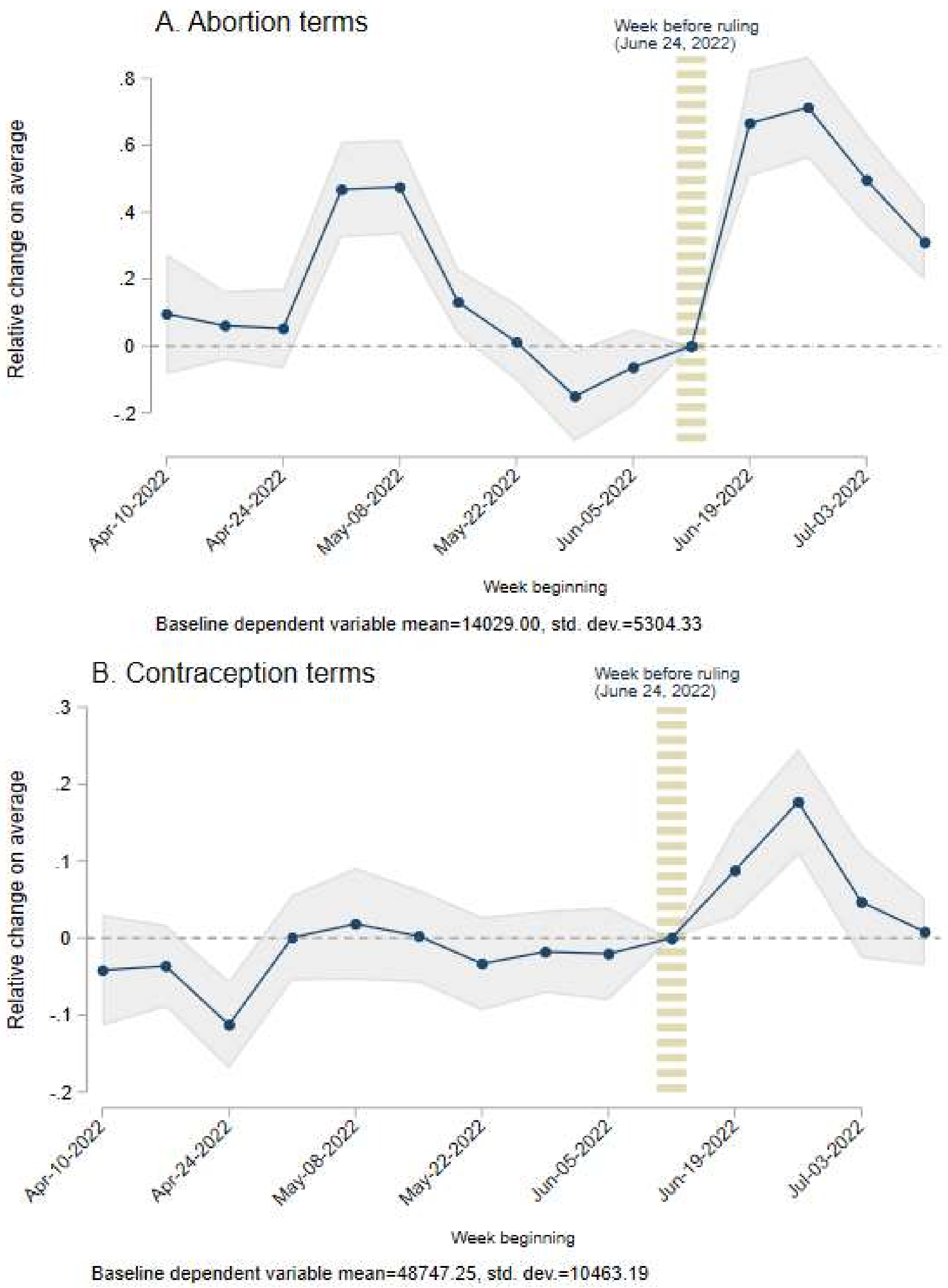

© 2023 Gupta et al. *JAMA Health Forum.*

eFigure 9. Time-varying effects of the Dobbs Leak and Official Ruling on internet searches for 'Planned Parenthood', in trigger/pre-Roe ban states vs in states with laws protecting abortion access

Notes: *The week prior to the treatment (leak) is set as a reference (normalized value of 0): light navy vertical bar. The week prior to the official ruling on June 24, 2022 is depicted using the darker navy vertical bar. In brown are the estimated coefficients (95% CI, gray band) in the Poisson model (differences in log expected counts of search relative to the period prior to the event). The average search frequency of each term per state per day is provided at the bottom of the panel.*

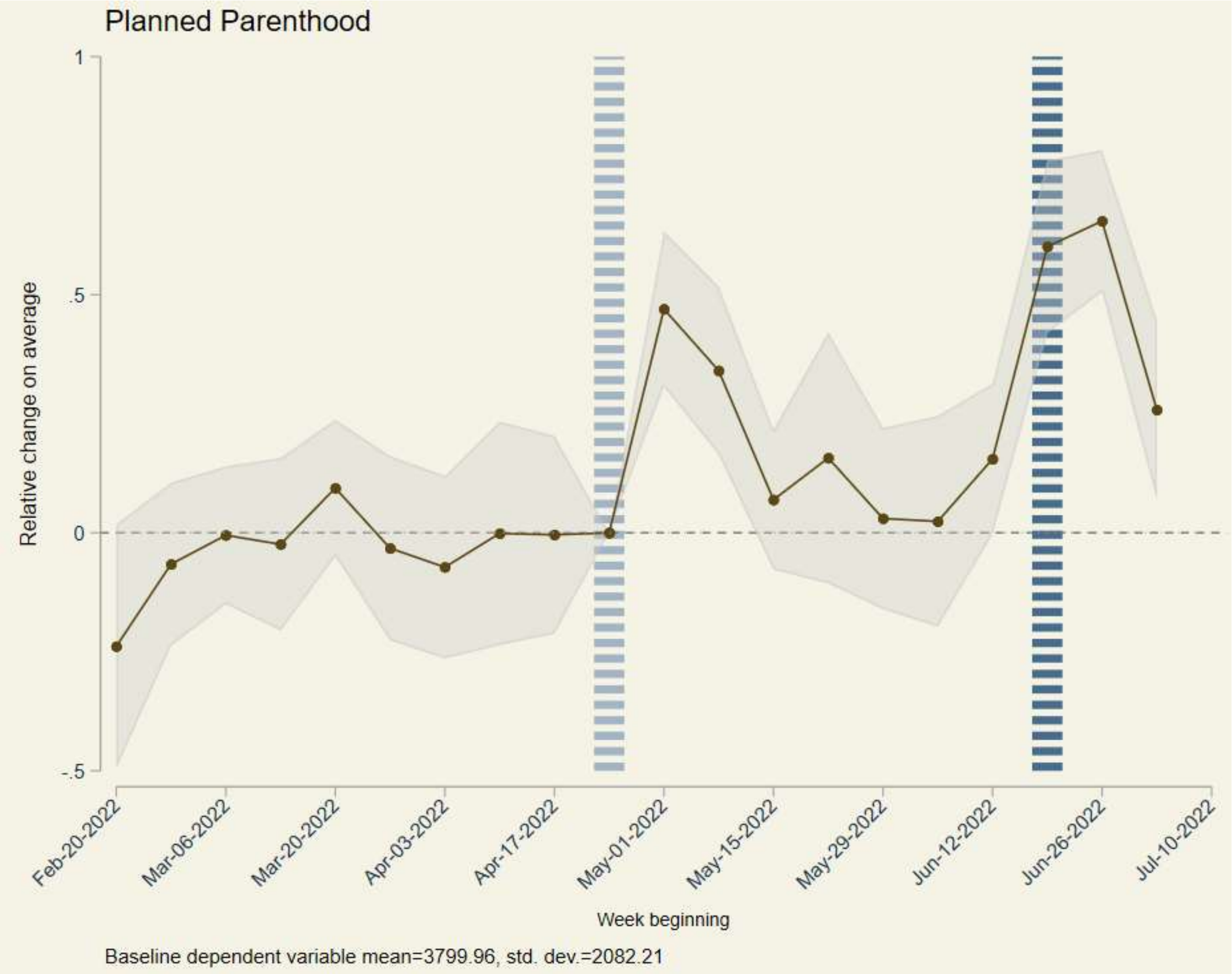

© 2023 Gupta et al. *JAMA Health Forum.*
