# Supplementary material for: Trends in Abortion- and Contraception-Related Internet Searches After the US Supreme Court Overturned Constitutional Abortion Rights: How Much Do State Laws Matter?
Source: JAMA Health Forum. 2023 Apr 28;4(4):e230518. doi: 10.1001/jamahealthforum.2023.0518 (PMC10148201; doi:10.1001/jamahealthforum.2023.0518)
Supplement: Supplement 2. — Data Sharing Statement [file jamahealthforum-e230518-s002.pdf]

Data Sharing Statement

Gupta. Trends in Abortion- and Contraception-Related Internet Searches After the US Supreme Court Overturned Constitutional Abortion Rights. *JAMA Health Forum*. Published April 28, 2023. doi:10.1001/jamahealthforum.2023.0518

Data
Data available: No

Additional Information
Explanation for why data not available: The search data for this study are available from Google Trends but are restricted in use; researchers may apply to Google Trends API for access. Replication code for the full analyses will be provided through a public GitHub repository.
